# Speciation of Lanthanide Metal Ion Dopants in Microcrystalline All-Inorganic Halide Perovskite CsPbCl_3_

**DOI:** 10.1021/jacs.3c11427

**Published:** 2024-03-28

**Authors:** Dominik J. Kubicki, Daniel Prochowicz, Albert Hofstetter, Amita Ummadisingu, Lyndon Emsley

**Affiliations:** a School of Chemistry, University of Birmingham, B15 2TT Birmingham, U.K.; b Institute of Physical Chemistry, Polish Academy of Sciences, Kasprzaka 44/52, 01-224 Warsaw, Poland; c Laboratory of Magnetic Resonance, Institute of Chemical Sciences and Engineering, Ecole Polytechnique Fédérale de Lausanne (EPFL), CH-1015 Lausanne, Switzerland; d Manufacturing Futures Laboratory, Department of Chemical Engineering, University College London, Torrington Place, WC1E 7JE London, United Kingdom

## Abstract

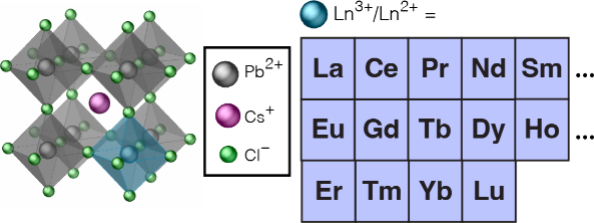

Lanthanides are versatile modulators of optoelectronic properties owing to their narrow optical emission spectra across the visible and near-infrared range. Their use in metal halide perovskites (MHPs) has recently gained prominence, although their fate in these materials has not yet been established at the atomic level. We use cesium-133 solid-state NMR to establish the speciation of all nonradioactive lanthanide ions (La^3+^, Ce^3+^, Pr^3+^, Nd^3+^, Sm^3+^, Sm^2+^, Eu^3+^, Eu^2+^, Gd^3+^, Tb^3+^, Dy^3+^, Ho^3+^, Er^3+^, Tm^3+^, Yb^3+^, Lu^3+^) in microcrystalline CsPbCl_3_. Our results show that all lanthanides incorporate into the perovskite structure of CsPbCl_3_ regardless of their oxidation state (+2, +3).

## Introduction

Lead halide perovskites have attracted significant attention as semiconducting materials for optoelectronic applications.^1^ Within this class of material, cesium lead halide (CsPbX_3_, X = Cl, Br, I) nanocrystals have been widely used for light emitting diodes (LEDs) and solar cell applications, offering a viable alternative to classical II–VI metal chalcogenides.^2−6^ Doping of CsPbX_3_ materials with metal ions is an effective way of tuning their optoelectronic response (emission wavelength and photoluminescence quantum yield) and stability. Rare earth metal ions, specifically lanthanides, have been particularly appealing as dopants owing to their sharp emission spectra which extend down to the near-infrared (NIR) region. This property makes them suitable for NIR and white LEDs, emitters, photodetectors, in addition to application in counterfeit security measures, optical temperature sensing, and optical data encoding.^7−15^ Various combinations of lanthanides have been introduced to halide perovskites, mostly in the form of nanocrystals, to tune their properties.^12,16−24^ These explorations build on the use of lanthanide doping in other classes of phosphor materials, typically oxides, silicates, and phosphates, which have been underway since at least the early 1940s when efficient luminescence of Eu^2+^ doped phosphors was observed under UV excitation.^25,26^ Halide perovskite phosphors doped with lanthanides have also recently been reported.^27^

Knowledge of the speciation of lanthanides in host matrices is essential to understand how they incorporate since the local structure of the dopant and its homogeneity within the host determine its optical properties.^28^ In the case of lanthanides with a partially filled 4f subshell, solid-state NMR^29^ has been particularly useful to study their incorporation into inorganic materials, including phosphors, because their paramagnetism leads to substantial changes in the NMR shifts and relaxation properties of the host.^30−35^ More generally, paramagnetic NMR techniques have been essential for studying atomic-level structure in areas such as battery materials,^36^ pharmaceuticals,^37^ metalloproteins,^38^ porous solids,^39^ optoelectronic materials,^40^ and polymers.^41,42^ Despite the growing use of solid-state NMR to determine speciation in MHPs^43^ and despite the importance of lanthanides in metal halide perovskites, their atomic-level speciation in metal halide perovskites has remained largely unexplored.^7^ Xiang et al. first explored lanthanide speciation in metal halide perovskites and found that Eu^2+^ has the capacity to incorporate into CsPbI_2_Br enhancing its efficiency in solar cells.^44^ Very recently, Xu et al. have used aberration-corrected atomic-resolution scanning transmission electron microscopy and three-dimensional atom probe tomography to directly observe Yb^3+^ inside CsPbCl_3_ nanocrystals.^24^

Here we focus our attention on the host material most widely used in light emission studies, cesium lead chloride, CsPbCl_3_.^45^ We set out to answer the following question: do all rare earth ions have the capacity to mix with CsPbCl_3_ at the atomic level? The answer to this question is not obvious since there are no rigorous rules for predicting the incorporation of dopants into halide perovskites and, more generally, the formation of solid solutions in extended solids. Empirical rules have been successful but have limited reliability in complex systems. Some prominent examples of phenomenological prediction of phase equilibria are the Hume–Rothery rules for multicomponent alloys,^46^ the computational CALPHAD methodology applicable to other types of solids,^47^ and the Goldschmidt tolerance factor for perovskites.^48,49^ Machine learning (ML)-based approaches are currently gaining prominence.^50^ While the question of lanthanide doping in CsPbCl_3_ has been explored for some lanthanides using long-range structure characterization techniques (XRD, TEM),^7,51^ dopants do not typically form periodic structures in the host matrix. Therefore, the complementary use of local structure characterization techniques is essential to identifying their location. A number of recent examples have shown that predicting the speciation of dopants in halide perovskites is nontrivial.^43^ For example, the incorporation of Co^2+^ into MAPbI_3_ (MA = methylammonium)^52^ was proposed but solid-state NMR investigations have shown that it is not the case.^53^ Grey et al. used paramagnetic effects in solid-state NMR to demonstrate the limited miscibility of Y_2_Sn_2_O_7_ and Nd_2_Sn_2_O_7_ pyrochlores.^30^ Finally, the speciation of potassium and guanidinium ions in hybrid halide perovskites had been subject to a long-standing debate that was settled using solid-state NMR.^54,55^

Here we determine a comprehensive understanding of the entire lanthanide dopant space in CsPbCl_3_ developed using ^133^Cs NMR. Unlike common materials characterization strategies, this technique offers the advantage of being sensitive to the structure topology, local symmetry, halide composition, and doping of the perovskite host.^43,56,57^ Paramagnetic rare earth metal ions are expected to induce paramagnetic relaxation enhancements (PRE) of the otherwise diamagnetic perovskite host matrix, leading to shortening of the ^133^Cs longitudinal relaxation times.^30,35,58^ In addition, the interaction between the unpaired 4f electrons of the lanthanide and the ^133^Cs nuclei may cause the resonance frequency of the latter to shift.^30,58^ The diamagnetic lanthanides (La^3+^ and Lu^3+^), if incorporated, may affect ^133^Cs shifts by inducing local lattice distortions.^43^ They may also affect ^133^Cs *T*_1_ relaxation, which in CsPbCl_3_ is driven by the modulation of dipolar couplings with the neighboring nuclei,^59^ because ^139^La and ^175^Lu are 99.9% and 97.4% abundant, respectively, and therefore are expected to couple magnetically with ^133^Cs.

Here, we exploit these ideas to understand the speciation of rare earth metal ions in this gold standard light emitting halide perovskite. We first report a facile solid-state route to microcrystalline CsPbCl_3_ doped with lanthanides (Ln^*n*+^ = Ce^3+^, Pr^3+^, Nd^3+^, Sm^3+^, Sm^2+^, Eu^3+^, Eu^2+^, Gd^3+^, Tb^3+^, Dy^3+^, Ho^3+^, Er^3+^, Tm^3+^, Yb^3+^, Lu^3+^) and then use cesium-133 solid-state NMR to demonstrate their successful incorporation into the perovskite structure.

## Experimental Section

### Materials

The following materials were used in this study: CsCl (Sigma, 99.999%), PbCl_2_ (Sigma, 99.999%, anhydrous), LaCl_3_ (Sigma, >99.99%, anhydrous), CeCl_3_ (Sigma, 99.99%, anhydrous), PrCl_3_ (Sigma, 99.99%, anhydrous), NdCl_3_ (AlfaAesar, 99.9%, anhydrous), SmCl_3_ (Sigma, 99.9%, anhydrous), SmI_2_ (Sigma, 99.9%, anhydrous), EuCl_3_ (Sigma, 99.99%, anhydrous), EuI_2_ (Sigma, 99.999%, anhydrous), GdCl_3_ (Sigma, 99.99%, anhydrous), TbCl_3_ (Sigma, 99.99%, anhydrous), DyCl_3_ (Sigma, 99.99%, anhydrous), HoCl_3_ (Sigma, 99.9%, anhydrous), ErCl_3_ (Sigma, 99.9%, ultra dry), ErI_3_ (abcr, 99.9%, anhydrous), TmCl_3_ (Sigma, 99.9%, anhydrous), YbCl_3_ (Sigma, 99.99%, anhydrous), LuCl_3_ (Sigma, 99.99%, anhydrous).

### Perovskite Mechanosynthesis

Starting materials were stored inside a glovebox in dry nitrogen at room temperature. Microcrystalline perovskite powders were synthesized by grinding the reagents in an electric ball mill (Retsch Mixer Mill MM-200) for 30 min at 25 Hz using an agate grinding jar (10 mL) and an agate ball (⌀10 mm). We used agate jars to ensure that the reaction proceeds to completion (Figure S1). The resulting powders were annealed at 300 °C for 2 min. The following amounts of reagents were used. Undoped CsPbCl_3_: CsCl (168 mg, 1 mmol), and PbCl_2_ (278 mg, 1 mmol). For CsPbCl_3_ doped with excess Ln^2+/3+^ ions, the following amounts of lanthanide halides were added to a mixture of CsCl (168 mg, 1 mmol) and PbCl_2_ (278 mg, 1 mmol): LaCl_3_ (24 mg, 0.1 mmol), CeCl_3_ (25 mg, 0.1 mmol), PrCl_3_ (25 mg, 0.1 mmol), NdCl_3_ (2.5, 25, and 50 mg, for 0.01, 0.1, and 0.2 mmol, respectively), SmCl_3_ (26 mg, 0.1 mmol), SmI_2_ (40 mg, 0.1 mmol), EuCl_3_ (26 mg, 0.1 mmol), EuI_2_ (41 mg, 0.1 mmol), GdCl_3_ (26 mg, 0.1 mmol), TbCl_3_ (27 mg, 0.1 mmol), DyCl_3_ (27 mg, 0.1 mmol), HoCl_3_ (27 mg, 0.1 mmol), ErCl_3_ (27 mg, 0.1 mmol), ErI_3_ (55 mg, 0.1 mmol), TmCl_3_ (28 mg, 0.1 mmol), YbCl_3_ (28 mg, 0.1 mmol), LuCl_3_ (28 mg, 0.1 mmol).

### NMR Measurements

Solid-state MAS NMR spectra of ^133^Cs (65.6 MHz at 11.7 T) and ^207^Pb (104.7 MHz at 11.7 T) were recorded on a Bruker Avance III 11.7 T spectrometer equipped with a 3.2 mm CPMAS probe. ^133^Cs shifts were referenced to a 1 M aqueous solution of cesium chloride, using solid CsI (δ = 271.05 ppm) as a secondary reference.^60^^207^Pb chemical shifts were referenced to liquid (CH_3_)_4_Pb using the chemical shift of Pb(NO_3_)_2_ as a secondary reference (δ_iso_ = −3494 ppm).^61^ For the saturation–recovery experiments, saturation was achieved by applying a train of 30 π/2 pulses spaced by 3 ms. All measurements were carried out using identical conditions, i.e., at 22 kHz MAS rate with the sample temperature of ∼54 °C measured using the linear shift dependence of ^79^Br on temperature (0.025 ppm/K) in solid KBr (Figure S16).^62^ For the quantitative spectra, a recycle delay of 500 s was used for undoped CsPbCl_3_, 200 s was used for the 10% doped materials, 60 s was used for the 20% Nd^3+^ doped material, and 5 s was used for Cs_4_NdCl_6_. The fast-recycling spectra were recorded with a recycling delay of 1 ms. Between 4 and 32 scans were used for the quantitative spectra and between 1024 and 256000 for the fast-recycling spectra. The link to all the raw data is https://zenodo.org/records/10853121. The saturation–recovery data were fitted in MATLAB using the stretched exponential function, and the fitted parameters are reported at the 95% confidence level (Supplementary Note 3).

### Powder X-ray Diffraction

Powder XRD patterns of the materials were recorded by using a Panalytical Empyrean diffractometer with a Cu target (Kα1 = 1.540 56 Å) using background-free holders made of monocrystalline silicon. Variable-temperature powder XRD was recorded using Bruker D8 Advance diffractometer equipped with a high-temperature oven chamber (Anton Paar, HTK 1200N) using a Cu target. The link to the raw data is https://zenodo.org/records/10853121.

### Scanning Transmission Electron Microscopy (STEM) Energy-Dispersive X-ray Spectroscopy (EDX)

STEM EDX measurements were carried out on a Tecnai Osiris microscope at 200 kV by using the STEM mode and Super-X detector to acquire EDX elemental maps.

## Results and Discussion

**Figure 1:**
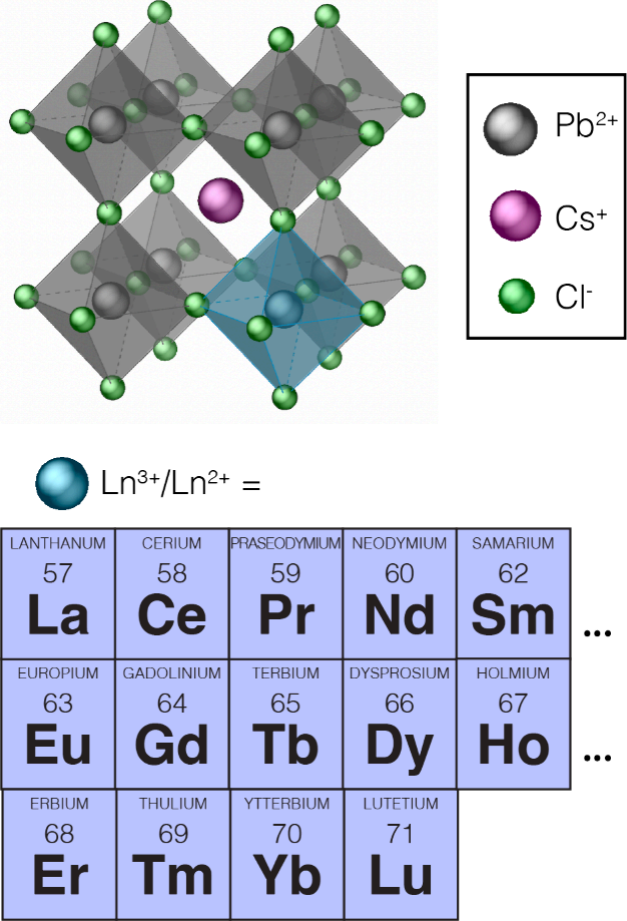
Schematic representation of the 3D perovskite structure of CsPbCl_3_ doped with lanthanides (Ln^*n*+^ = La^3+^, Ce^3+^, Pr^3+^, Nd^3+^, Sm^3+^, Sm^2+^, Eu^3+^, Eu^2+^, Gd^3+^, Tb^3+^, Dy^3+^, Ho^3+^, Er^3+^, Tm^3+^, Yb^3+^, and Lu^3+^).

Figure 1 schematically shows the materials prepared in this study. Lanthanide-doped CsPbCl_3_ was prepared by solid-state mechanosynthesis with the addition of 10 mol % excess of rare earth chlorides with the lanthanide in the +3 oxidation state. In addition, in the cases of Sm and Eu, iodides with the lanthanide in the +2 oxidation state were also used. The materials were annealed to remove grinding-induced defects, and XRD showed that in all cases, essentially phase-pure perovskite structure has formed (Figure S2). The XRD peak positions were essentially unaffected within the measurement uncertainty relative to undoped CsPbCl_3_. CsPbCl_3_ exhibits three structural phase transitions near room temperature. The material is cubic above 47 °C (phase I), tetragonal between 47 and 42 °C (phase II), orthorhombic between 42 and 37 °C (phase III), and monoclinic below 37 °C (phase IV). Each of these phases is expected to have a slightly different ^133^Cs shift. To obtain a meaningful comparison, it was essential to carry out the NMR experiments under identical conditions as spinning and gas flow inside the probe may affect the temperature of the sample and therefore the signal position (Figure S3). The sample temperature inside the rotor measured using the ^79^Br shift of solid KBr was ca. 54 °C in all the measurements carried out, and as such, the material was in the cubic phase. XRD carried out on selected materials at 55 °C confirms that the presence of the dopants does not affect the crystal structure appreciably under the conditions of the MAS NMR experiment. Since the incorporation of dopants is expected to affect ^133^Cs resonance frequencies and the *T*_1_ relaxation of the corresponding species, we recorded ^133^Cs spectra and saturation–recovery data for all of the materials.

**Figure 2:**
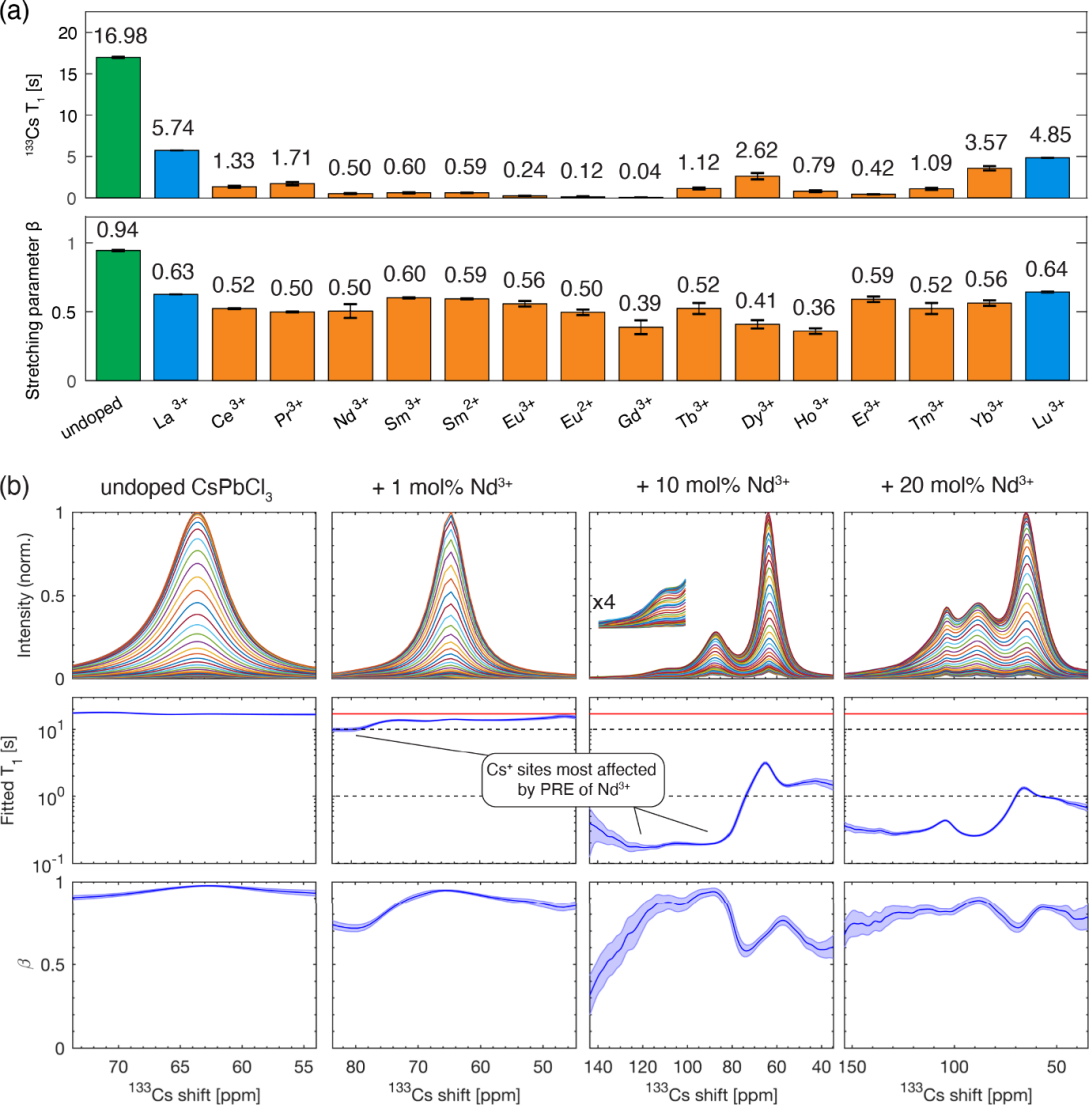
^133^Cs longitudinal relaxation time measurements in CsPbCl_3_ doped with lanthanides. (a) Fitted longitudinal relaxation times, *T*_1_, and stretching parameters, β, obtained for a fit to the integral of the whole spectrum. The fitting was carried out using the stretched exponential function (Supplementary Note 1). (b) Distribution of the fitted *T*_1_ and β values across the spectrum for different Nd^3+^ loadings (0, 1, 10, and 20%). The top panels show an overlay of the saturation–recovery spectra for different recovery times.

We first focus on the effect of lanthanide doping on the ^133^Cs *T*_1_ relaxation. Figure 2 shows the ^133^Cs *T*_1_ values measured for undoped and doped CsPbCl_3_ using a saturation–recovery sequence with the integral being taken across the whole spectrum. The saturation–recovery curves were fitted using a stretched exponential because a distribution of *T*_1_ values is expected (see Supplementary Note 1 for details).^53^ We observe *T*_1_ shortening for all dopants, indicating that they modify the parent host structure. We rationalize the effect of the diamagnetic lanthanides (La^3+^ and Lu^3+^) on ^133^Cs *T*_1_ by invoking the dipolar relaxation mechanism of ^133^Cs in CsPbCl_3_.^59^ In the doped structures, the ^133^Cs nuclei exhibit additional dipolar couplings to the nearly 100% abundant ^139^La and ^175^Lu nuclei, and we expect that the modulation of these dipolar couplings leads to the experimentally observed ∼3 times faster *T*_1_ relaxation. As expected, the *T*_1_ shortening in the case of paramagnetic lanthanides is more substantial (5 times faster for Yb^3+^ and 425 times faster for Gd^3+^) and related to the PRE effect. This effect is caused by the through-space dipolar interaction between the ^133^Cs nuclei and the unpaired 4f electrons. Efficient nuclear relaxation occurs as a result of the modulation of this interaction at frequencies on the order of the ^133^Cs Larmor frequency (104 MHz at 11.7 T, corresponding to correlation times on the order of 10 ns), and since the strength of this interaction scales as 1/*r*^6^, it is a direct local probe of the presence of the paramagnetic species within the host structure. In all cases the recovery is stretched, indicating that there is a distribution of *T*_1_ values within the material.^63^ The stretching factor, β, is 0.6 for Lu^3+^ and La^3+^, and it is 0.4–0.6 for paramagnetic lanthanides. For a host matrix with randomly distributed paramagnetic dopants and in the absence of spin diffusion, it has been shown theoretically and experimentally that the stretching parameter β takes on a value of 0.5.^63−65^ This result is a strong indication that (a) for 10 mol % Ln^3+^ doping, the lanthanides are randomly distributed within the host matrix, and (b) ^133^Cs–^133^Cs spin diffusion is essentially absent. While ^133^Cs is 100% abundant, we conclude that its low gyromagnetic ratio and the relatively large intercesium spacings renders spin diffusion negligible on the relaxation time scale here.^66^ Because there is a distribution of local environments with distinct *T*_1_ values, we next show how those values vary across the inhomogeneously broadened spectrum for Nd^3+^ when its content increases from 0 to 20 mol % (Figure 2b).

The *T*_1_ of undoped CsPbCl_3_ is uniform across the entire peak with β ≈ 1 (β decreases slightly on both sides of the signal possibly because these regions might contain contributions from grain boundaries, which are known to exhibit faster relaxation,^67^ hence the slightly broader distribution of *T*_1_ values in these regions). For 1 mol % Nd^3+^, while there is very little change to the line shape, it is clear that the *T*_1_ decreases across the resonance, with the local environments at higher frequencies being affected the most. The β value for those environments decreases to ∼0.7. A spectrum recorded using a very short recycle delay enhances the intensity of these fast-relaxing environments and shows that there is a new local Cs^+^ environment already present at this low doping ratio (Figure S4). For 10 mol % Nd^3+^, there is a substantial drop in *T*_1_ across the entire line shape, and two distinct peaks appear at higher frequencies relative to the initial peak. These signals manifestly correspond to Cs^+^ sites near Nd^3+^ because of the substantial PRE associated with them. The main peak is now fully affected by PRE, which means that all Cs^+^ sites within the material sense the presence of Nd^3+^ through the 1/*r*^6^ relationship (as spin diffusion is negligible on this time scale for ^133^Cs, as mentioned above).^66^ This result is expected based on the binomial law:^68^ for 10 mol % Nd^3+^, the probability of encountering a Cs^+^ site with 0 Nd^3+^ nearest neighbors in a 10 Å radius is less than 1%, assuming a random dopant distribution. This result therefore also indicates that there is no substantial Nd^3+^ clustering, which would lead to the presence of Nd^3+^ deficient regions unaffected by PRE within the material, in agreement with the interpretation of the experimentally observed β = 0.5.

Interestingly, while the average β for this sample is 0.5 (Figure 2a), the spectrally resolved β fitting in Figure 2b shows that there is a distribution of β values across the line shape. We interpret this result as follows: because each spectral region corresponds to Cs^+^ sites at a different distance from the paramagnetic dopant, the local distribution of *T*_1_ for each spectral region is slightly different, leading to different β values. For 20 mol % Nd^3+^, the PRE increases still further, with the *T*_1_ being less than 1 s over nearly the entire line shape. It is noteworthy that β takes on values between 0.7 and 0.8 across the spectrum in this material, which again corroborates the conclusion that there is no ^133^Cs–^133^Cs spin diffusion. Efficient spin diffusion in a material with such high concentration of paramagnetic dopants would lead to β = 1.^69^ We have carried out an analogous analysis for the other materials doped with paramagnetic lanthanides, and in all cases the new peaks are strongly affected by PRE (see the Supporting Information section “Data fitting results”). It is worth noting that there is a continuum of apparent *T*_1_ values on going from the low frequency side of the spectrum (weaker PRE) to the high frequency side (stronger PRE). The gradual change in the apparent *T*_1_ indicates that each of the peaks is a convolution of many local environments with slightly different distances to the paramagnetic ion, and as a result, the spectrum is inhomogeneously broadened. Finally, we also note that intermediate β values (0.5 < β < 1) have also been observed in some materials (for average β, i.e. fitted for the whole spectrum simultaneously), such as silicon carbide.^70^ Those cases have been interpreted in terms of the inhomogeneous distribution of paramagnetic impurities. Taken together, the ^133^Cs *T*_1_ relaxation analysis shows that all the lanthanide ions incorporate into the host matrix and, for 10 mol % Ln^3+^ doping, are randomly distributed. This result is corroborated by the STEM EDX maps of selected materials, which show that the distribution of Cs and the lanthanides is highly uniform within the perovskite grains (Figures S20–22). Our results agree with those in a recent study by Xu et al., who used 3D atom probe tomography to study an Yb-doped CsPbCl_3_ single crystal and concluded that the dopant distribution in their material also is highly uniform.^24^

**Figure 3:**
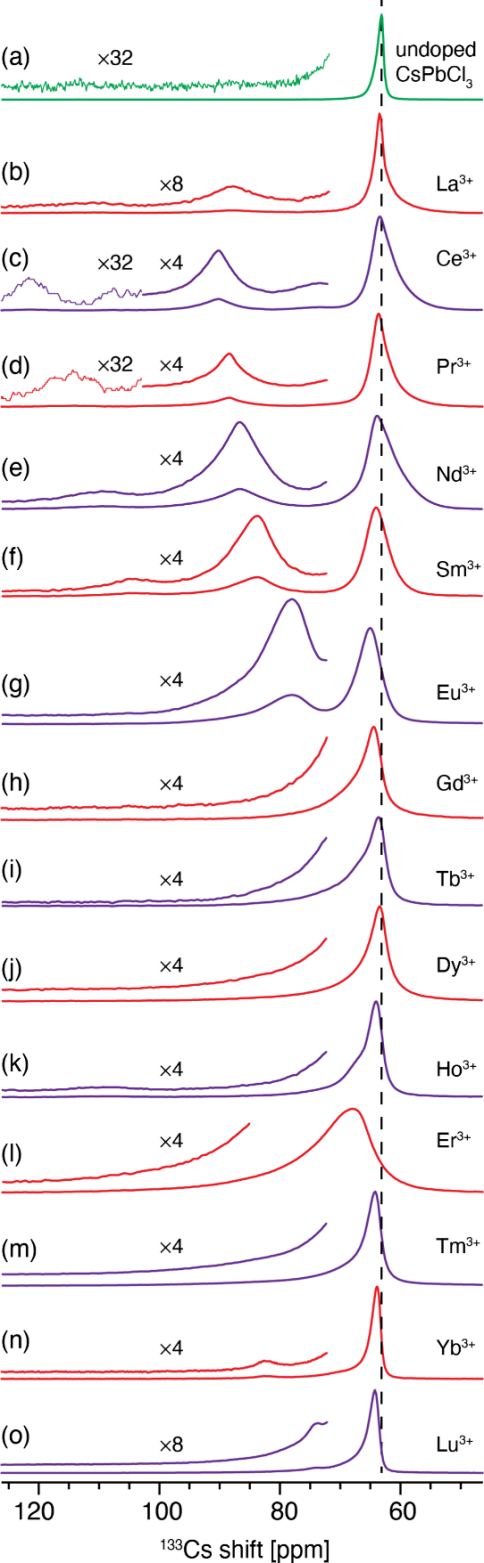
Quantitative ^133^Cs MAS NMR spectra of bulk mechanochemical CsPbCl_3_ perovskite compositions: (a) undoped and (b–o) doped with 10 mol % excess LnCl_3_ (Ln^3+^ given next to each spectrum).

We next analyzed the quantitative ^133^Cs spectra (Figure 3). The ^133^Cs signal of undoped CsPbCl_3_ is narrow (full width at half maximum, FWHM, ∼85 Hz, 1.3 ppm) and nearly symmetrical. When CsPbCl_3_ is doped with 10 mol % lanthanide ions, the main peak visibly broadens (see Figure S5 for the fitted values), and for some dopants, additional signals corresponding to new local environments appear in the quantitative spectrum. In the f^0^–f^5^ (La^3+^–Sm^3+^) group, two peaks are apparent that shift to lower frequencies as the number of f electrons increases. In the f^6^ ion (Eu^3+^), they overlap, leading to a single apparent peak. In the f^7^–f^9^ (Gd^3+^–Dy^3+^) and f^11^–f^12^ (Er^3+^–Tm^3+^) groups, there are no additional peaks, while a low-intensity peak is visible in Ho^3+^ (f^10^), Yb^3+^ (f^13^) and Lu^3+^ (f^14^). The signal of the Er-doped material is substantially broader and shifted to higher frequencies relative to all of the other materials. The inspection of this data set allows us to draw the following conclusions: (a) the addition of rare earth ions in the +3 oxidation state to CsPbCl_3_ leads to changes in the atomic-level structure of the host material and, in conjunction with the PRE effects shown above, evidences aliovalent incorporation of the rare earth ions into CsPbCl_3_; (b) new local environments appear that are characterized by substantially shortened *T*_1_ values and these correspond to Cs^+^ sites in close proximity of the Ln^3+^ dopant; (c) the spectral changes are qualitatively the same for La^3+^ (f^0^, diamagnetic) and Ce^3+^–Eu^3+^ (f^1^–f^6^) suggesting that they have a common origin which is related to local structural changes rather than paramagnetic effects. This last distinction is subtle but important because paramagnetic doping is well-known to lead to large shifts that qualitatively resemble those seen here. We discuss this point in more detail now.

Paramagnetic (or hyperfine, hf) shifts arise due to two major mechanisms: (a) overlap between the unpaired electron density and the s orbitals of the NMR nucleus (referred to as contact or Fermi shift) and (b) through-space electron–nuclear magnetic dipole interaction (pseudocontact shift, PCS). Since Fermi shifts require orbital overlap, they are common in materials where the paramagnetic ion is covalently bonded to the NMR nucleus, for example in lanthanide pyrochlores, phosphates, and actinide oxides.^58,71−73^ They are also present when the NMR nucleus has an ionic bond but is close enough to the paramagnetic species for an orbital overlap to occur. This is common in transition metal compounds, for example, Cs_2_CuCl_4_,^74^ Cs_2_CoCl_4_,^75^ CsMnCl_3_,^76^ or Prussian blue analogues such as CsNi[Co[CN]_6_].^77^ In lanthanide-doped CsPbCl_3_, the most likely location for the lanthanide is the B-site, corresponding to aliovalent Pb^2+^ substitution with the concomitant formation of a Cs^+^ vacancy, because of the similarity of the ionic radii of Pb^2+^ (119 pm) and Ln^3+^ (86–103 pm). Conversely, aliovalent replacement of Cs^+^ (188 pm) with the much smaller lanthanide is unlikely. In the cubic phase of CsPbCl_3_, the distance between the Cs^+^ and Pb^2+^ sites is 485 pm and between two Cs^+^ sites 560 pm. This hypothesis has recently been corroborated by atomic-scale STEM EDX mapping of Yb-doped CsPbCl_3_ which evidenced that Yb^3+^ has the capacity to replace Pb^2+^, in addition to occupying interstitial sites within the perovskite structure.^24^ Considering the ionic radii of Cs^+^ (188 pm) and Ln^3+^ (86–103 pm) and the limited spatial extent of the 4*f* orbitals, transfer of unpaired electron density from Ln^3+^ to Cs^+^ is essentially impossible, regardless of the exact location and type of lanthanide. Indeed, Fermi shifts for lanthanides are typically on the order of thousands of ppm and, for example, in rare earth stannates, span the range between −4000 and +4000 ppm depending on the type of lanthanide ion (see Figure S6 for an example of a hf-shifted ^133^Cs spectrum).^71^

**Figure 4:**
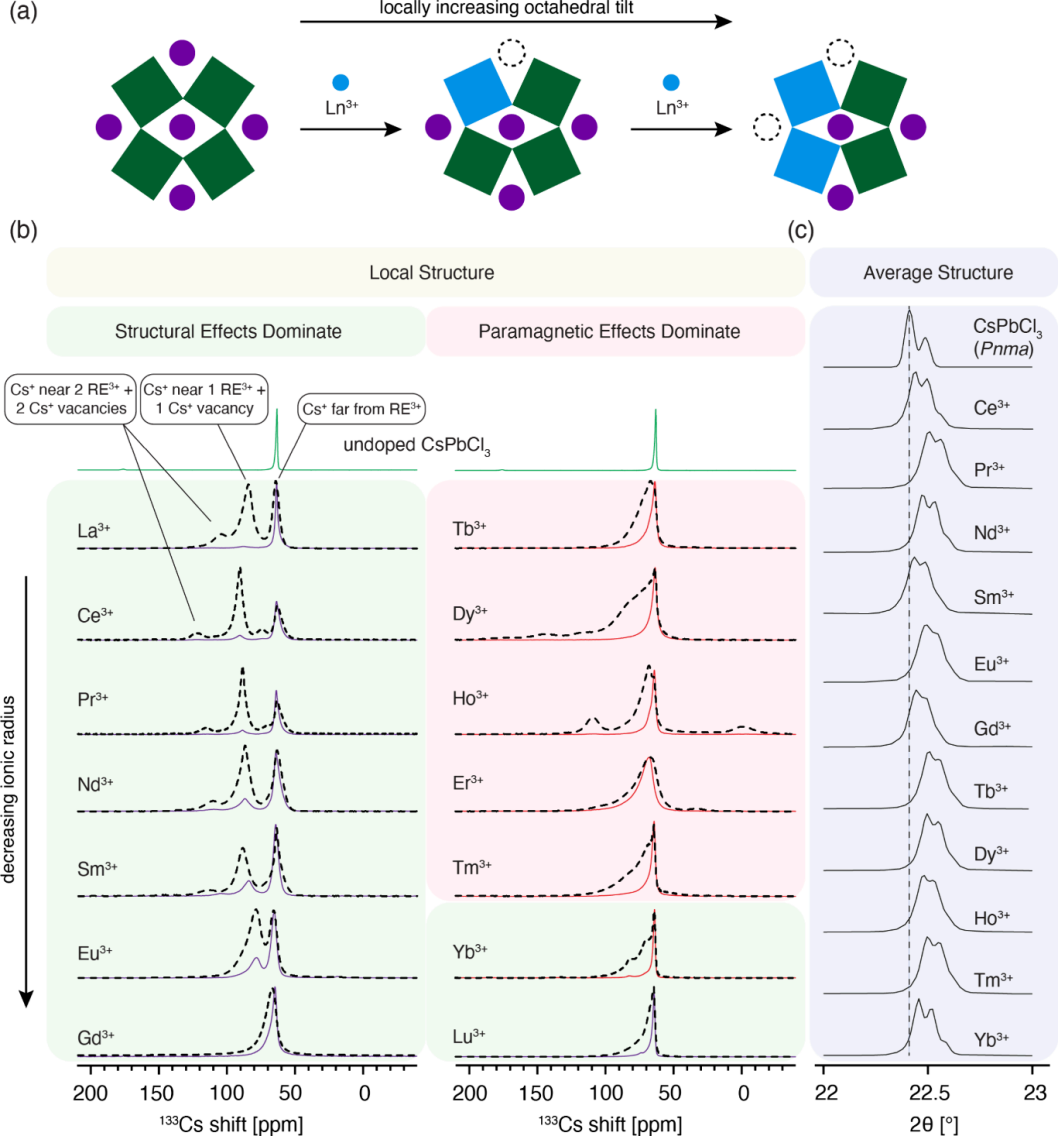
(a) Interpretation of the structural effect leading to the appearance of new ^133^Cs resonances in CsPbCl_3_ doped with lanthanides from La^3+^ to Gd^3+^. The decreasing ionic radius locally tunes the tolerance factor, leading to a gradual increase in the octahedral tilt. (b) Comparison of quantitative (solid lines) and fast-recycling (dashed lines) ^133^Cs MAS NMR spectra of the materials (a larger version of the figure is given in Figure S15). (c) Powder XRD patterns of selected materials (magnified [121] reflection). The small relative shift of the peak position in the different materials is attributed to experimental uncertainty rather than changes in the average structure.

Pseudocontact shifts result when the paramagnetic lanthanide has an anisotropic magnetic susceptibility, which depends on the symmetry of the local coordination environment. In CsPbCl_3_, the local environment is highly symmetric when Ln^3+^ occupies the B-site in the cubic phase (octahedral ligand field), which translates to vanishingly small PCS. The magnitude of PCS further depends on the Cs-RE distance, the angle θ between the RE magnetic axis of symmetry and the Cs-RE internuclear axis, and the type of lanthanide through its total angular momentum and *g* factor. Notably, the geometrical dependence of PCS contains a scaling factor of (3 cos^2^ θ – 1) which amounts to zero or nearly zero when θ = 54.74°, as is the case in the cubic or nearly cubic phase of CsPbCl_3_ (Figure S7). We can further exclude the possibility that the new signals appearing in the Ce–Gd group are caused by pseudocontact shifts by comparing the relative magnitudes of the shifts expected for the respective rare earth metal ions on theoretical grounds with those observed experimentally (Figure S8). The relative magnitudes are in clear disagreement (by a factor of 12 for Sm^3+^ and a factor of 2 for Pr^3+^ and Eu^3+^, scaled to the shift of Ce^3+^) evidencing that PCS is not the origin of these shifts. An analogous analysis can be done for Fermi shifts, and it also predicts magnitudes which are off by up to a factor of 5 and therefore in disagreement with the data. Finally, Gd^3+^ (f^7^) is a special case since it has a half-full 4f shell and as such has a PCS of strictly 0.^35^ These results unambiguously indicate that in the La–Gd group of dopants the spectra are dominated by structural rather than paramagnetic effects. In fact, the separation between the original peak of CsPbCl_3_ and the two new local environments steadily decreases in the La–Gd series in a nearly linear fashion (Figure S9). The key structural parameter that changes in this series is the ionic radius, which decreases as the 4f shell is filled, an effect known as lanthanide contraction. This result suggests that the position of the new peaks is related to the ionic radius of the dopant and may be a proxy of the local octahedral distortion (Figure 4a). Specifically, substituting the smaller lanthanide (86–103 pm) for the larger Pb^2+^ (119 pm) with the concomitant formation of a Cs^+^ vacancy (ionic radius of Cs^+^: 188 pm) locally increases the octahedral tilting depending on the ionic radius of the lanthanide. That said, the average structure of the materials measured by X-ray diffraction at room temperatures remains monoclinic for all of the materials, indicating that this structural effect operates only in the close proximity of the dopant (Figure 3c).

We used the binomial theorem to calculate the probabilities for different local Cs environments as a function of the lanthanide content and compared them to the experimental peak areas for the two most intense peaks in the quantitative ^133^Cs spectra. The binomial theorem implies random distribution of the dopants across all the Pb sites. We found that the binomial prediction is in good qualitative agreement for all of the early lanthanide dopants (La^3+^, Pr^3+^, Ce^3+^, Sm^3+^, Eu^3+^, 1–20% Nd^3+^) (Figures S18 and S19). We note, however, that the binomial prediction slightly overestimates the population of the Pb_7_Ln local environment relative to that in the experiment. This can be rationalized by considering that the materials studied here are nonstoichiometric (aliovalent substitution, i.e., Pb^2+^ replaced by Ln^3+^) and the dopants (LnCl_3_) are added as excess relative to stoichiometric CsPbCl_3_. The second major source of discrepancy is likely the peak deconvolution process used to quantify the peak areas, especially in the case of Eu^3+^ where the peaks overlap substantially.

The situation is different in the Tb–Yb group, in which the expected relative magnitude of PCSs is 5–10 times larger than for the early lanthanides, owing to their substantially larger total angular momentum. In conjunction with nonideal cancellation of the geometrical factors described above, this may lead to detectable PCS. Because the pseudocontact interaction is anisotropic, the first indication that PCSs may be present is the appearance of many spinning sidebands in the spectrum. This effect is indeed evident for Tb, Dy, Ho, Er and Tm which have more pronounced spinning sidebands than the other lanthanides (Figures S10 and S11, 22 kHz MAS; Figure S12, 5 kHz MAS). That said, while detectable, this effect is relatively small as typical pseudocontact shift anisotropies for Ln^3+^ ions are on the order of thousands of ppm.

For example, the following values have been previously reported: 1500 ppm for Yb_2_Sn_2_O,^78^ 600 ppm (YbPO_4_), 2800 ppm (ErPO_4_), and 1700–4000 ppm in other rare earth metal orthophosphates.^79^ Here, the anisotropy is at most 200–400 ppm, although a precise fit is not possible due to the additional susceptibility anisotropy effect^78^ which makes the spinning sideband manifold a convolution of the two effects.

The signals affected by PCSs can be readily detected because PREs shorten substantially the recovery time of the local environments in the proximity of the rare earth metal ions. Their signals can therefore be enhanced by recording the spectra using short recycling delays. Figure 4b shows a comparison of the quantitative and fast-recycling spectra for each material. While for La^3+^-Gd^3+^, the qualitative picture is the same in both experiments, there are multiple additional signals visible in the Tb^3+^-Yb^3+^ group with fast recycling. In the case of Ho^3+^ and Er^3+^ they are present on either side of the original CsPbCl_3_ peak, a tell-tale sign of PCSs, whose relative sign depends on the (3cos^2^ θ – 1) factor and can be either positive or negative depending on the local geometry (see Figure S7). The relatively large shift and broadening of the Er^3+^ material relative to all the other lanthanides (Figure 3l) is likely caused by anisotropic bulk magnetic susceptibility broadening (ABMS) (See Supplementary Note 2 and Figure S23 for a more detailed discussion).^80,81^

We next show that incorporation also occurs for lanthanides in the +2 oxidation state (Sm^2+^ and Eu^2+^). To that end, we used SmI_2_ and EuI_2_ as the lanthanide ion precursors. The PRE effect in these samples is analogous to that seen for the Ln^3+^ ions (Figure 2), confirming that changing the oxidation state does not affect the capacity of these ions to incorporate into CsPbCl_3_. The corresponding ^133^Cs spectra are qualitatively similar between the two oxidation states, with one (for Eu^2+^) and two (for Sm^2+^) extra peaks formed upon doping, the only difference being a small shift to higher frequencies of all the peaks in the case of the +2 dopants caused by the concomitant iodide–chloride mixing (Figure S13).

While ^133^Cs is the most convenient local structure probe in CsPbCl_3_, for the sake of completeness, we also explored the effect of lanthanide doping on the ^207^Pb spectra in the case of Nd^3+^ (Figure S14). The spectrum has two distinct overlapping components, one broad and one narrow, corresponding to local Pb^2+^ environments that are affected by the paramagnetic dopant to a higher and lesser degree, respectively. The distribution of *T*_1_ values across the line shape corroborates this interpretation, with the broad part of the spectrum having a 20–50% shorter *T*_1_ than the narrow part. Notably, the ^207^Pb *T*_1_ relaxation data sets take considerably longer to acquire (∼12 h, compared to 2–4 h for ^133^Cs). Considering the time requirements, higher signal-to-noise ratio, and higher resolution of the ^133^Cs data sets, we conclude that ^133^Cs should be the nucleus of choice for studying paramagnetic effects in cesium halometalates.

In conclusion, we have shown that all of the rare earth metal ions have the capacity to incorporate into the perovskite structure of CsPbCl_3_, in both the +3 and +2 oxidation states.

We found that the incorporation of lanthanides leads to paramagnetic NMR effects detectable in the ^133^Cs NMR signal of the perovskite phase. Those effects include the appearance of additional signals associated with Cs^+^ sites in close proximity of the lanthanide, in addition to substantial shortening of the ^133^Cs longitudinal relaxation times, *T*_1_. Faster relaxation is also observed for incorporation of the diamagnetic lanthanides, La^3+^ and Lu^3+^, where we attribute it to enhanced dipolar relaxation induced by the ^139^La and ^175^Lu nuclear spins. In the case of the paramagnetic lanthanides, the 5–425 times faster *T*_1_ is caused by paramagnetic relaxation enhancement due to the through-space dipolar interaction between the unpaired 4f electrons and the ^133^Cs nuclear spins. In all cases, there is a nonuniform distribution of *T*_1_ values across the spectrum, which is strongly dependent on the lanthanide ion loading.

Our analysis indicates that the nearly linear changes in the ^133^Cs shifts observed for doping with the lanthanides from La^3+^ through Gd^3+^ are dominated by structural rather than paramagnetic effects. We suggest they are determined by the radius of the lanthanide ion rather than its magnetic properties, and therefore are a proxy of the local octahedral distortion.

On the other hand, for the lanthanides between Tb^3+^ and Tm^3+^, paramagnetic effects dominate, and we attribute the changes in the ^133^Cs NMR spectrum to pseudocontact shifts. We find no evidence of contact (Fermi) shifts on ^133^Cs, indicating that there is no significant overlap of the unpaired 4f electron spin density with the Cs^+^ site.

One of the important future areas of study will be the use of computational methods to fully assign ^133^Cs MAS NMR spectra of paramagnetically doped halide perovskites. These computations are expected to be demanding, owing to the need for fully relativistic treatment and inclusion of spin–orbit coupling. In the context of aliovalent doping, the key challenge will be to identify the possible structural models: Are the chloride ions involved in charge compensation? Do the vacancies cause a local distortion of symmetry leading to multiple possible inequivalent lanthanide positions? Answering these questions will go a long way toward understanding aliovalent doping in this class of solids.

Our work shows that solid-state NMR of metal halide perovskites doped with lanthanides is a highly sensitive indicator of the dopant incorporation, loading, and interaction with the perovskite structure. We expect the framework developed here to aid in atomic-level characterization of optoelectronic materials with this remarkably diverse and technologically significant class of dopants.

## Data Availability

The raw NMR and XRD data are available on Zenodo: https://zenodo.org/records/10853121.

## Supporting Information Available

XRD, additional NMR analysis, additional NMR spectra, and relaxation data (PDF)
