# Supplementary material for: Speciation of Lanthanide Metal Ion Dopants in Microcrystalline All-Inorganic Halide Perovskite CsPbCl3
Source: J Am Chem Soc. 2024 Mar 28;146(14):9554–63. doi: 10.1021/jacs.3c11427 (PMC11009948; doi:10.1021/jacs.3c11427)
Supplement: Supplementary file 1 — ja3c11427_si_001.pdf [file ja3c11427_si_001.pdf]

# Supplementary Information

## Speciation of Lanthanide Metal Ion Dopants in Microcrystalline All-Inorganic Halide Perovskite $CsPbCl_3$

Dominik J. Kubicki,*[a] Daniel Prochowicz,[b] Albert Hofstetter,[c] Amita Ummadisingu,[d] Lyndon Emsley*[c]

[a]*School of Chemistry, University of Birmingham, B15 2TT, Birmingham, UK*
[b]*Institute of Physical Chemistry, Polish Academy of Sciences, Kasprzaka 44/52, 01-224 Warsaw, Poland*
[c]*Laboratory of Magnetic Resonance, Institute of Chemical Sciences and Engineering, Ecole Polytechnique Fédérale de Lausanne (EPFL), CH-1015 Lausanne, Switzerland*
[d]*Manufacturing Futures Laboratory, Department of Chemical Engineering, University College London, Torrington Place, WC1E 7JE, London, United Kingdom*

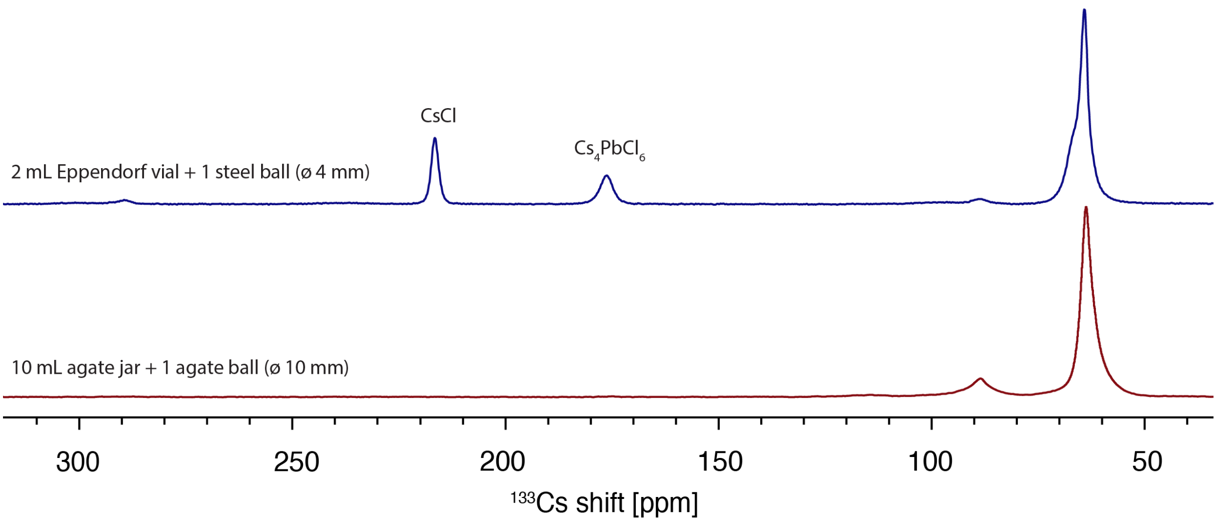


Figure S1. $^{133}$Cs MAS NMR spectra of a $CsPbCl_3$ doped with a 10 mol% excess of $PrCl_3$, prepared using mechanosynthesis using (a) a polypropylene Eppendorf vial and a stainless steel ball (⌀ 4 mm) and (b) an agate grinding jar (10 ml) and an agate ball (⌀10 mm). The resulting powders were annealed at 300 °C for 2 minutes. In contrast to the agate jar, the use of the polypropylene vial leads to a not fully reacted mixture of phases.

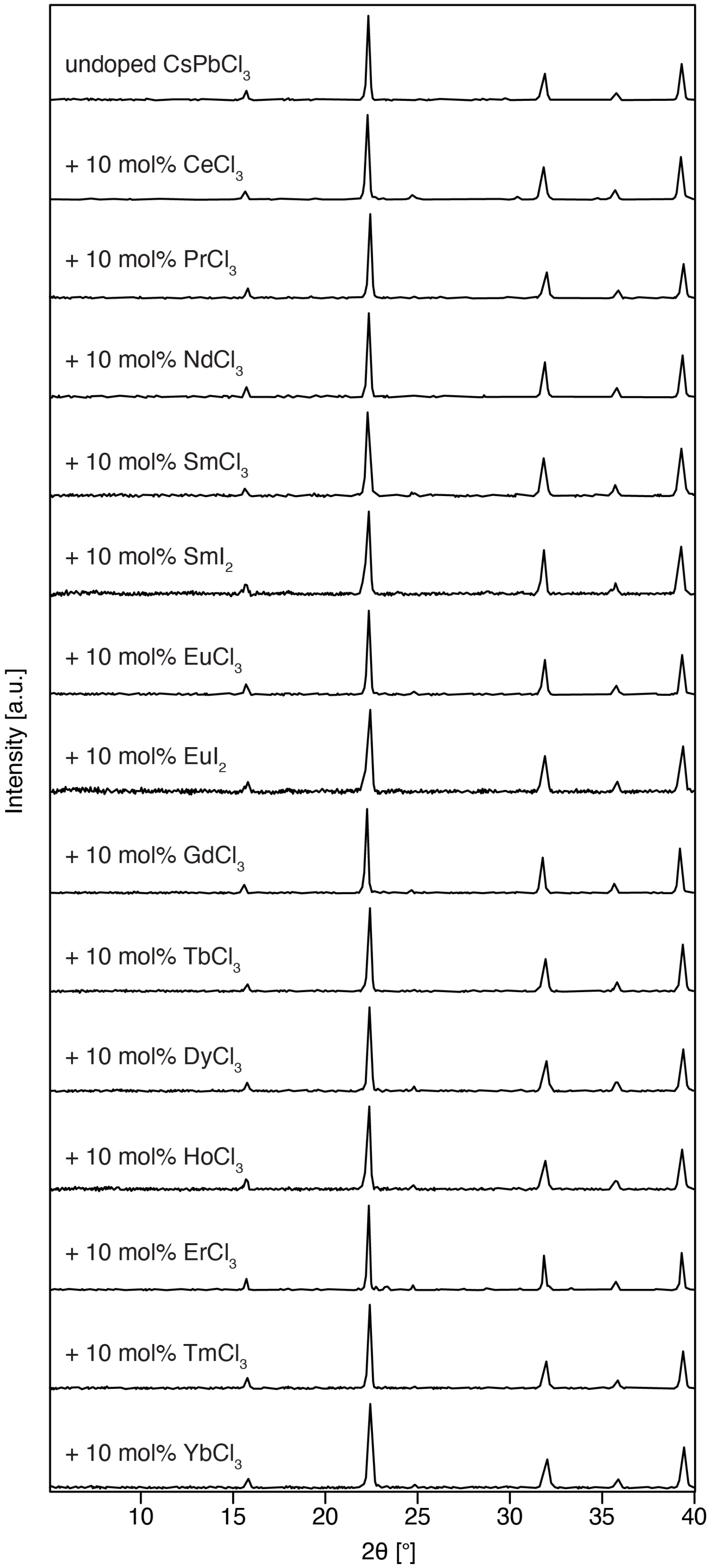


Figure S2. Powder XRD patterns of the materials.

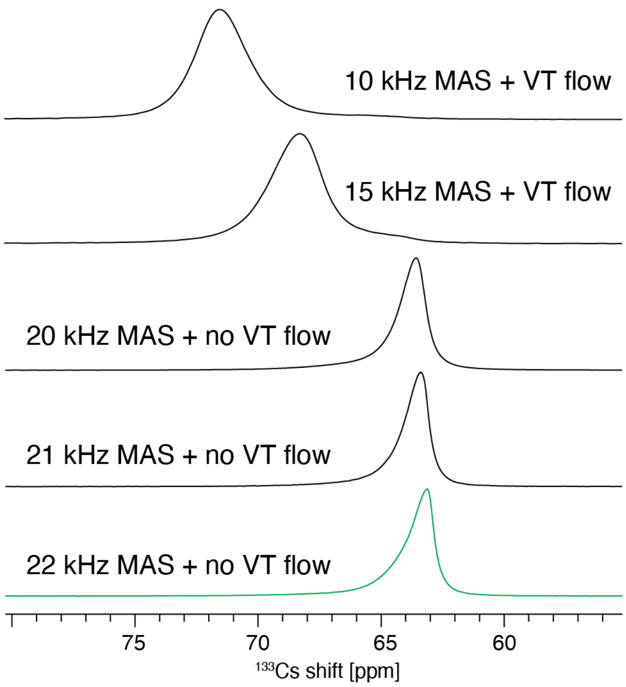


Figure S3. The effect of the probe gas flow (VT) and spin rate on the $^{133}$Cs signal position in undoped $CsPbCl_3$.

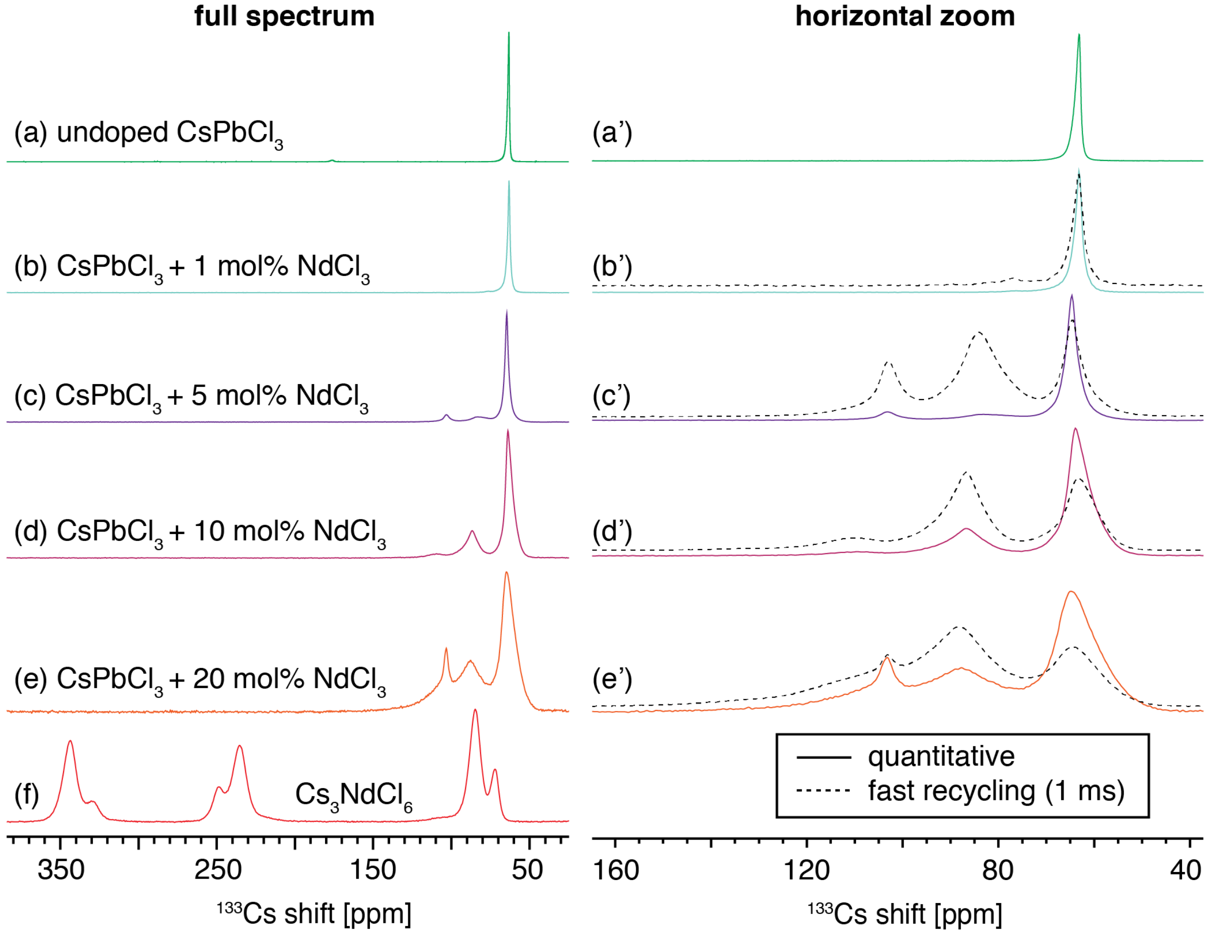


Figure S4. Comparison of quantitative (solid lines) and fast-recycling (dashed lines) $^{133}$Cs MAS NMR spectra of the materials doped with different amounts of $NdCl_3$.

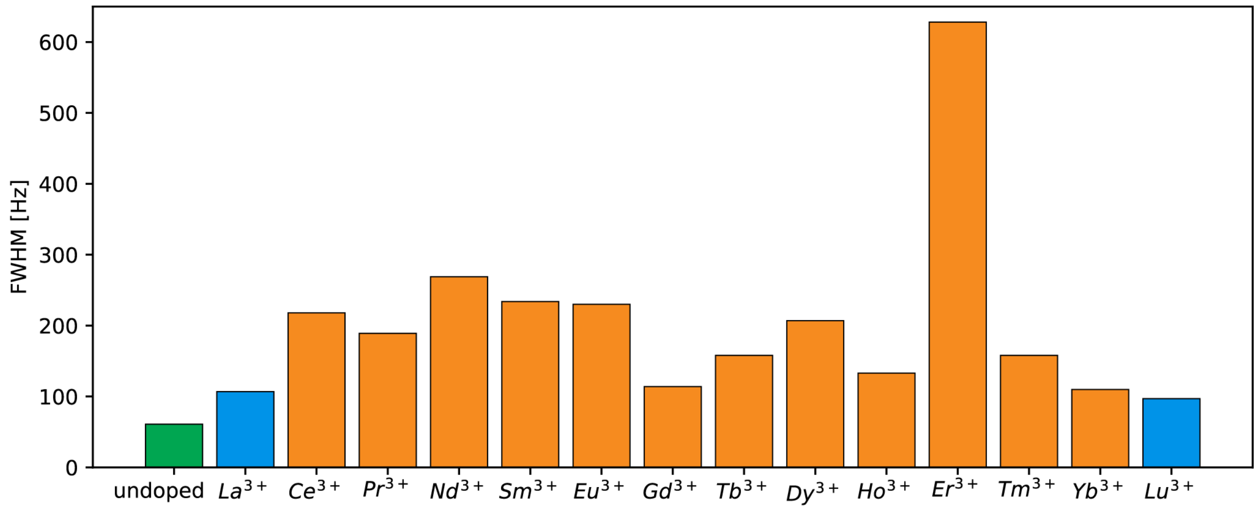


Figure S5. Fitted full width at half maximum (FWHM) of the most intense peak in the quantitative $^{133}$Cs spectra of the materials.

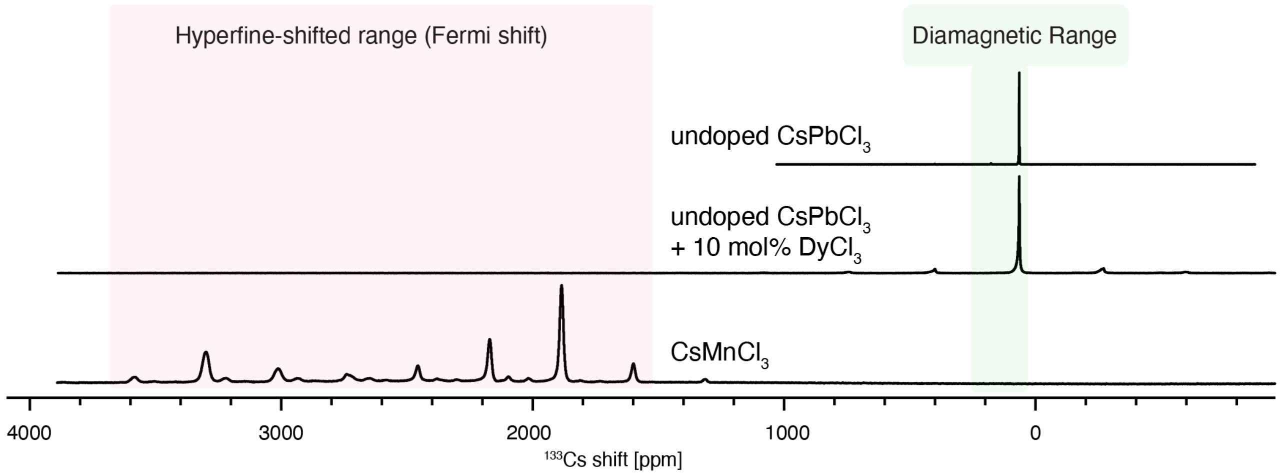


Figure S6. An example of a $^{133}$Cs spectrum shifted due to Fermi contact interaction (unpaired electron spin density overlapping with the s orbitals of $^{133}$Cs) in $CsMnCl_3$. The diamagnetic range is also shown for reference.

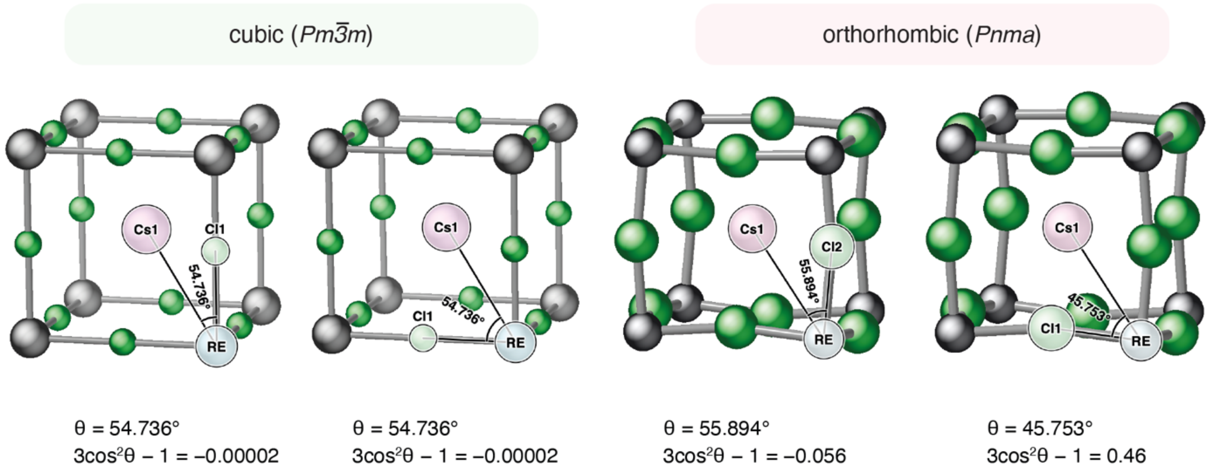


Figure S7. The geometrical dependence of pseudocontact shifts calculated for cubic and orthorhombic $CsPbCl_3$. RE (rare-earth ion) = $Ln^{3+}$. The dependence contains a scaling factor of $(3\cos^2\theta - 1)$ which amounts to zero or nearly zero when θ=54.74° as is the case in the cubic or nearly cubic phase of $CsPbCl_3$. The local symmetry could be locally distorted in the presence of a $Ln^{3+}$ and a $Cs^+$ vacancy leading to a non-vanishing PCS. The magnetic axis of symmetry is assumed to be aligned with the RE-halide bond (highest symmetry direction).

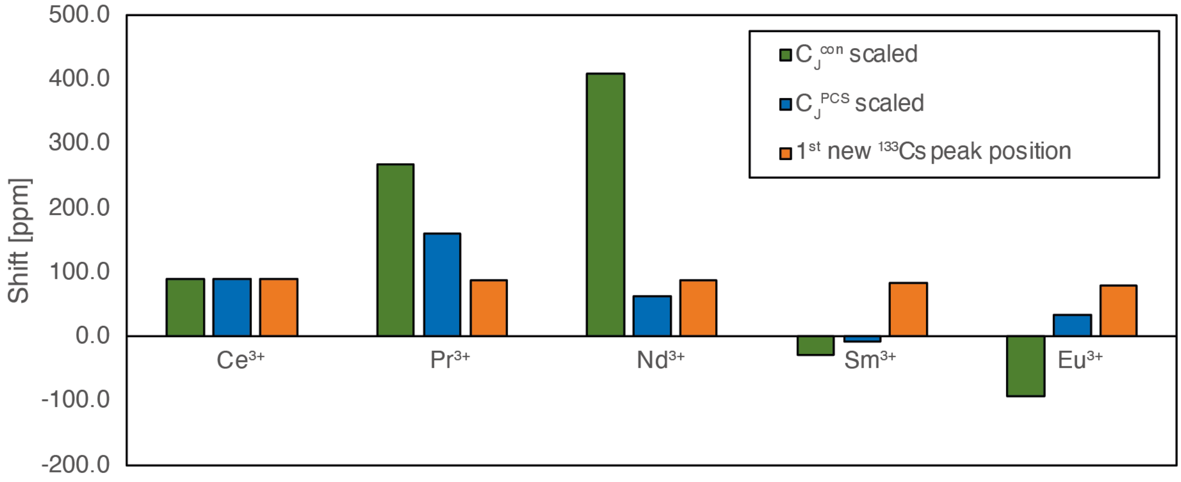


Figure S8. Predicted magnitude of the contact (Fermi) and pseudocontact (PCS) shifts. The contact shift is proportional to $C_J^{con}$,and the PCS is proportional to $C_J^{PCS}$. These parameters have been scaled relative to the position of the 1st extra peak in the $Ce^{3+}$ doped material. If one of the contributions was solely responsible for the observed shift of the 1st extra peak in the case of other lanthanides, one would expect the corresponding scaled values of $C_J^{con}$ or $C_J^{PCS}$ to match the experimental shift, which is not the case. This result indicates that the extra peaks observed in these samples are related to changes in structure rather than paramagnetic effects. The values of $C_J^{con}$ or $C_J^{PCS}$ have been evaluated at 300 K by Pell et al.[1]

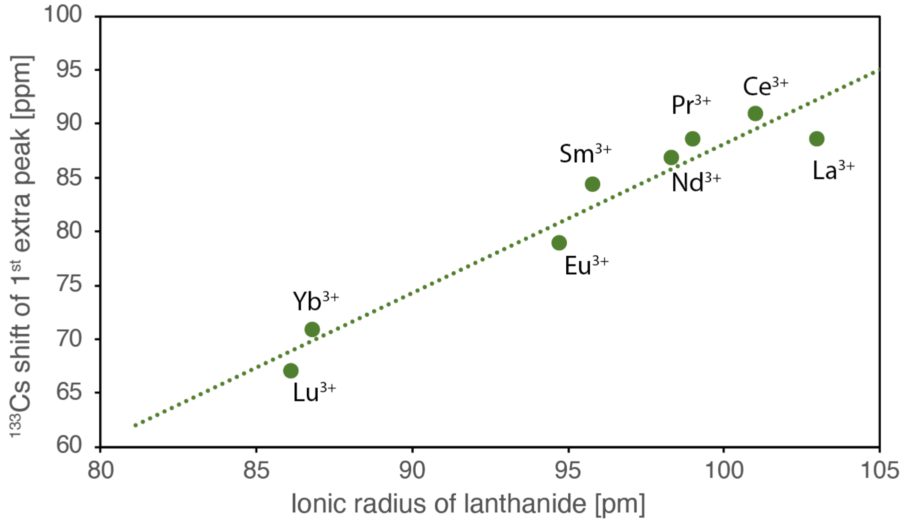


Figure S9. Position of the 1st extra peak in the $^{133}Cs$ spectra of $CsPbCl_3$ doped with the lanthanides from $La^{3+}$ to $Eu^{3+}$, and $Yb^{3+}$ and $Lu^{3+}$. The nearly linear correlation between the lanthanide ion radius and the extra peak position suggest that the shift is determined by local structure (degree of octahedral tilt) rather than paramagnetic effects.

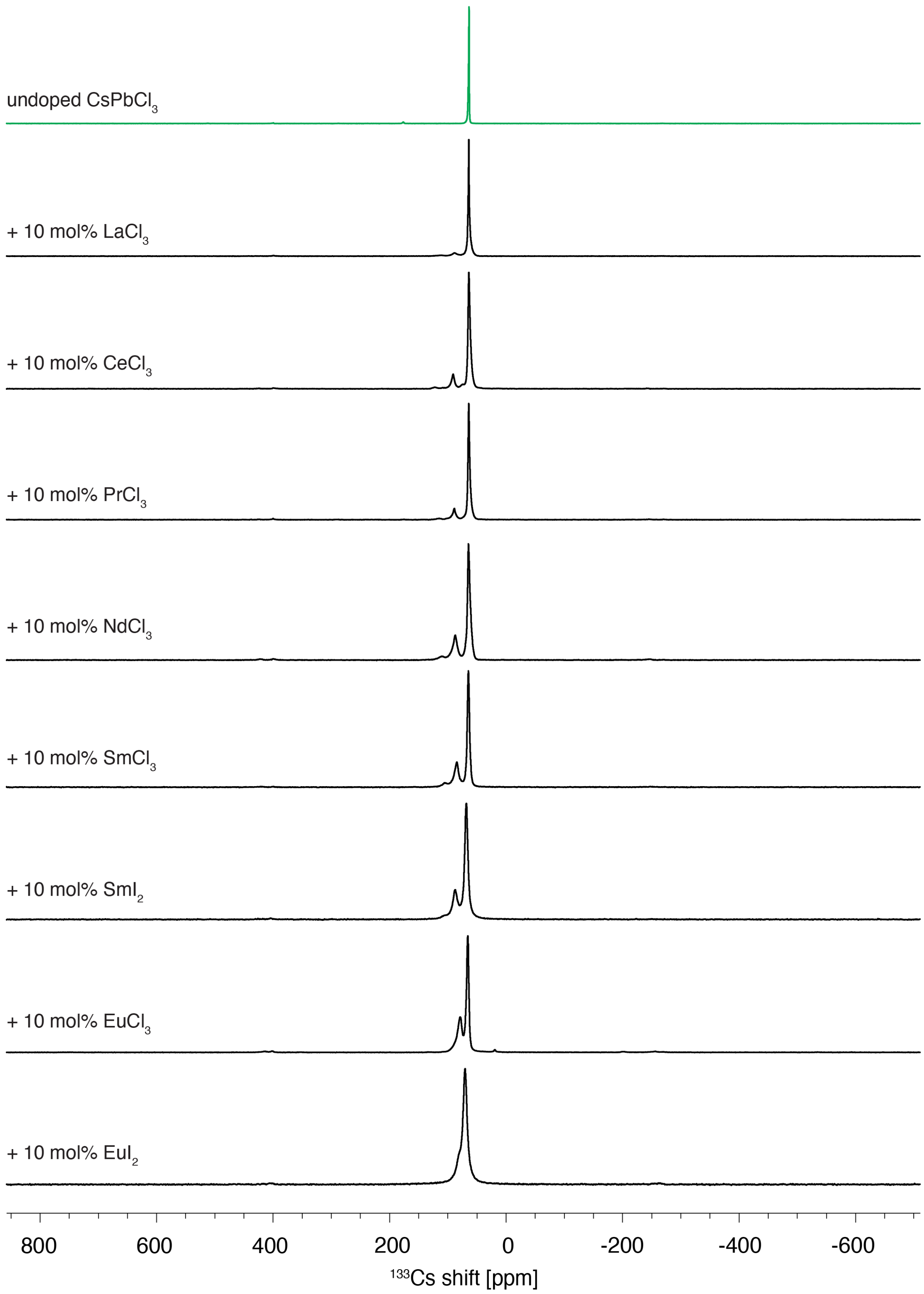


Figure S10. Full quantitative $^{133}$Cs MAS NMR spectra of the materials (22 kHz MAS).

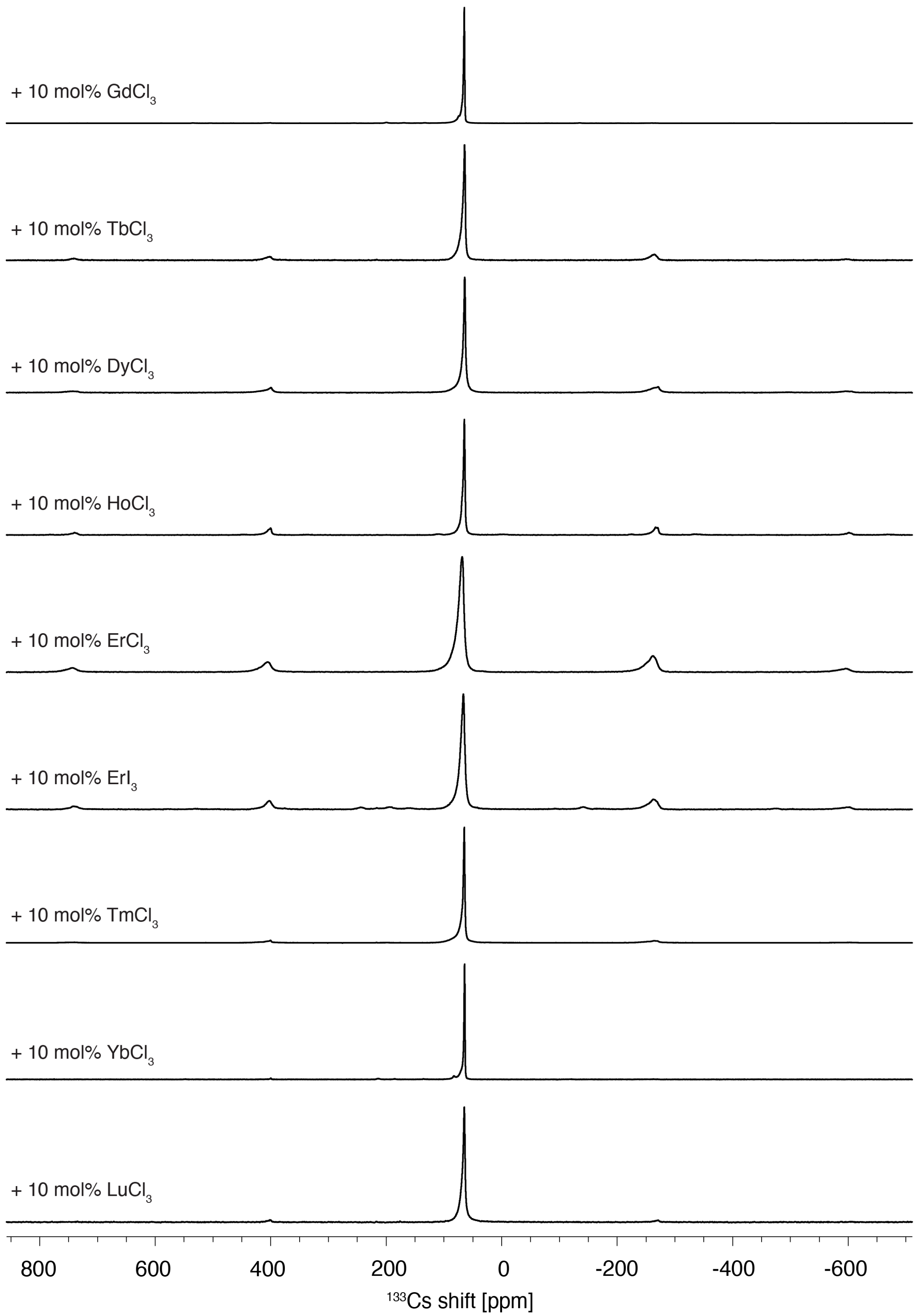


Figure S11. Full quantitative $^{133}Cs$ MAS NMR spectra of the materials (22 kHz MAS) (continued).

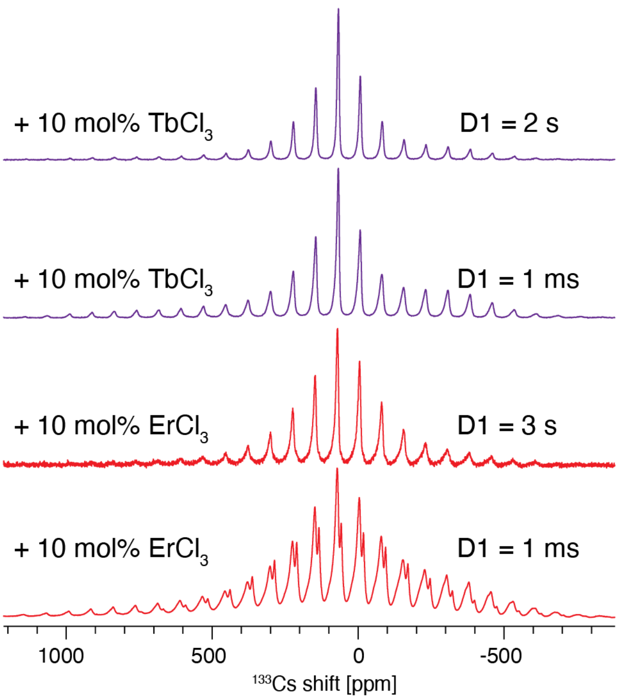


Figure S12. $^{133}Cs$ MAS NMR spectra recorded at 5 kHz MAS to probe the PCS anisotropy. The fitted PCS anisotropy magnitude is at most 200-400 ppm but may be limited by susceptibility anisotropy, as discussed in the main text.

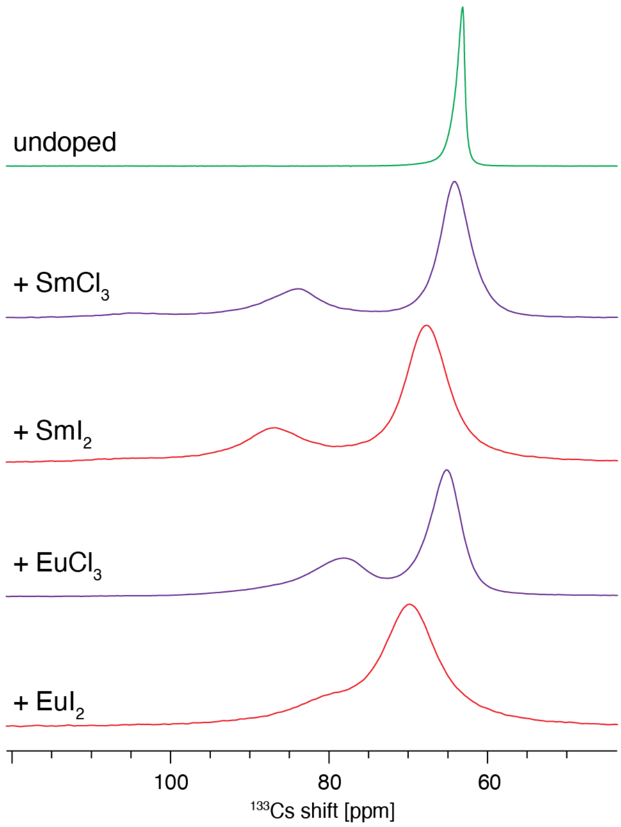


Figure S13. $^{133}Cs$ MAS NMR spectra of the materials doped with Eu and Sm ions in the +2 and +3 oxidation states. While the qualitative picture is similar for the two oxidation states, there is a relative shift attributed to iodide-chloride mixing, since the +2 precursors were iodides.

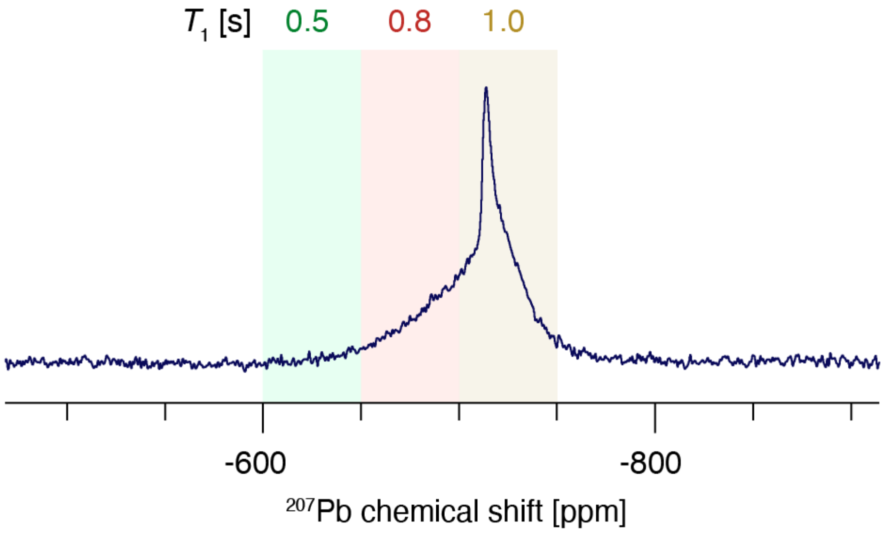


Figure S14. $^{207}$Pb MAS NMR spectrum of $CsPbCl_3$ + 10 mol% $NdCl_3$. Recycle delay: 5 s, number of scans: 1024. The fitted $T_1$ values of the different spectral ranges are shown.

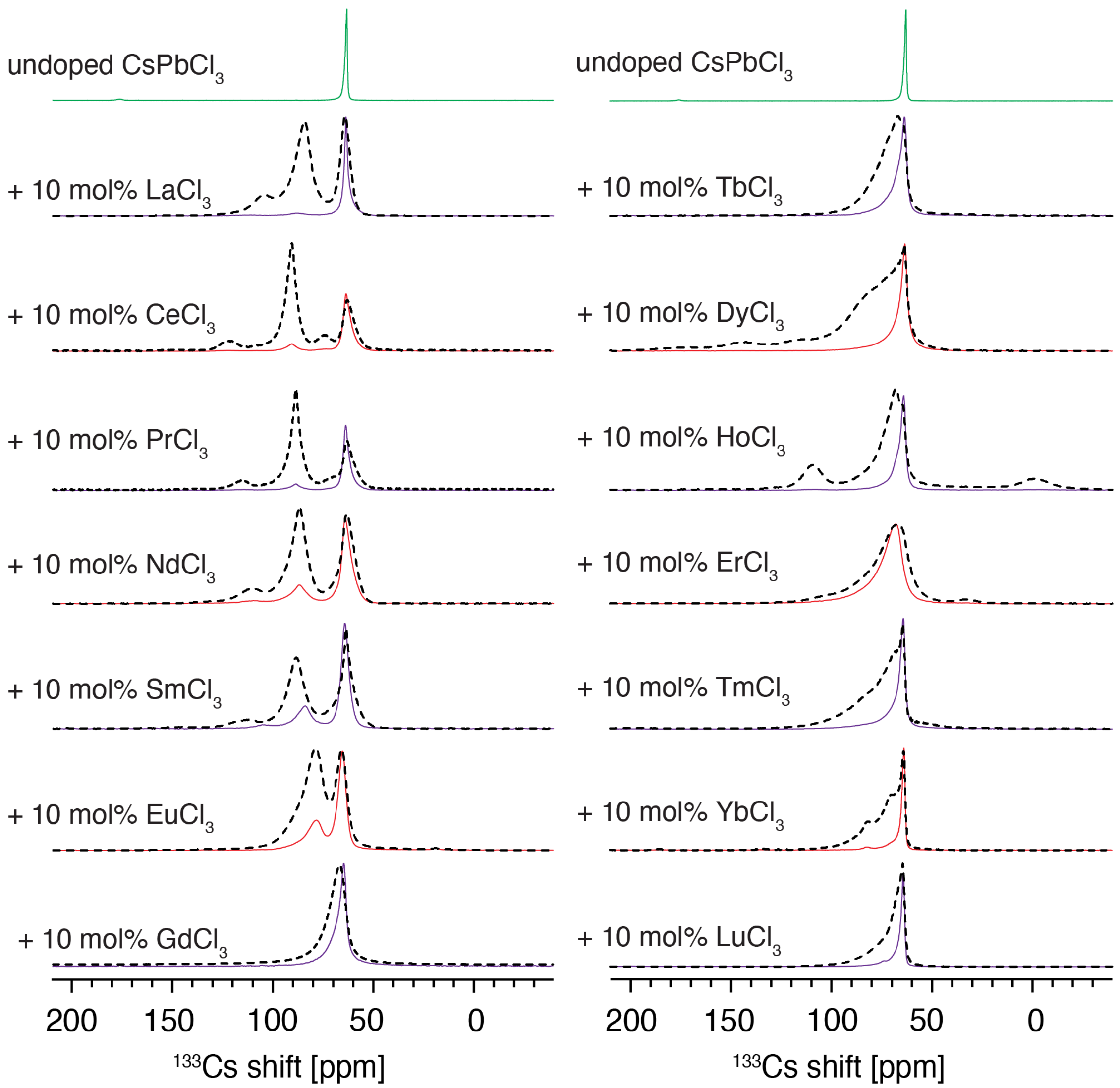


Figure S15. Comparison of quantitative (solid lines, recycle delay of 200s) and fast-recycling (dashed lines, recycle delay of 1 ms) $^{133}$Cs MAS NMR spectra of the materials.

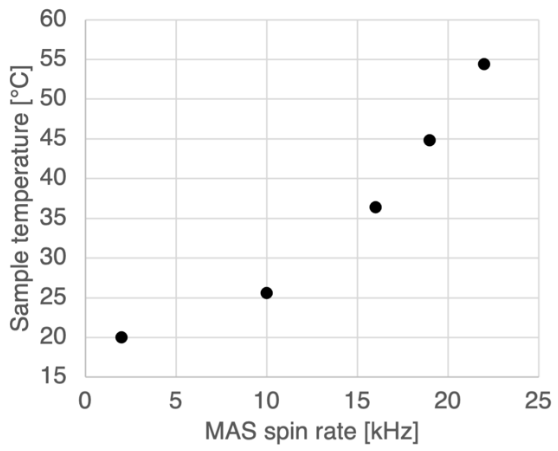


Figure S16. Effective sample temperature using the linear shift dependence of $^{79}Br$ on temperature (0.025 ppm/K) in solid KBr (10.1016/j.jmr.2008.09.019).

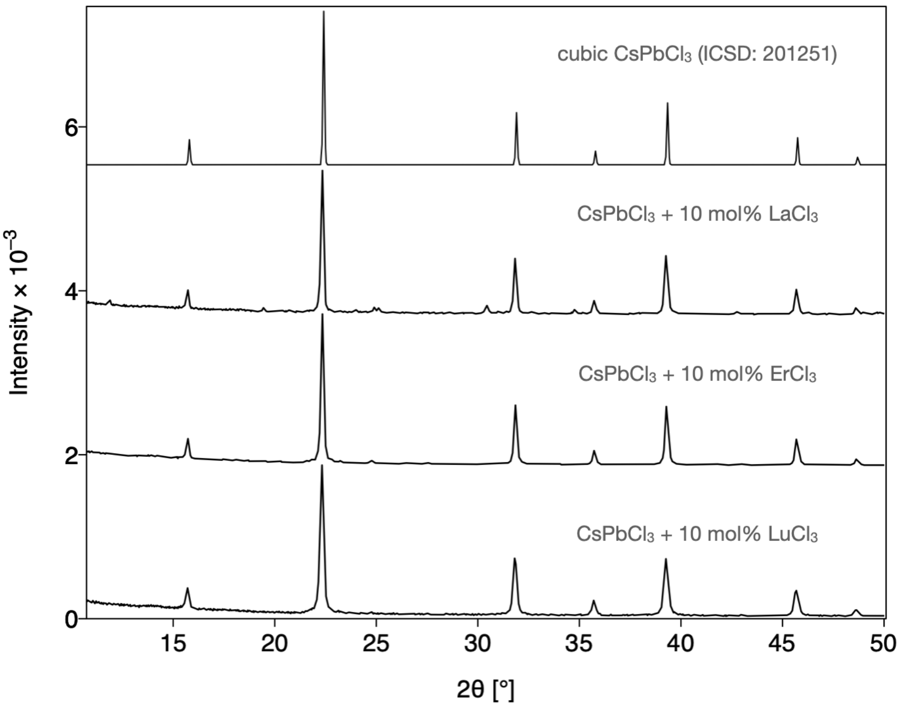


Figure S17. Powder XRD patterns of selected materials recorded at 55 °C.

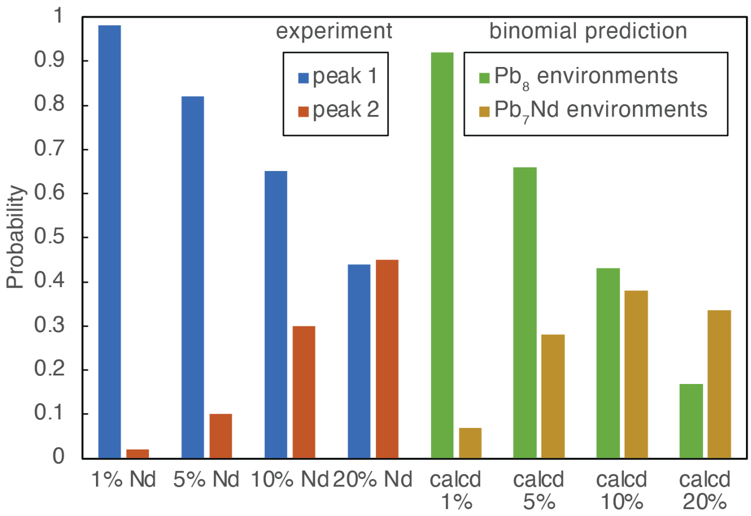


Figure S18. Comparison of (left part) the experimentally measured areas of the two most intense peaks in the quantitative $^{133}Cs$ spectra of $NdCl_3$-doped $CsPbCl_3$ and (right part) the probabilities of occurrence of different local Cs environments calculated using the binomial theorem. The two local Cs environment shown here are: Cs surrounded by 8 Pb atoms ($Pb_8$), and Cs surrounded by 7 Pb and 1 Nd atoms ($Pb_7Nd$). The experimental areas were obtained by fitting the spectra. Note that the materials studied here are non-stoichiometric (aliovalent substitution, i.e., $Pb^{2+}$ replaced by $Nd^{3+}$) and the dopant ($NdCl_3$) was added as excess relative to stoichiometric $CsPbCl_3$, which is the likely origin of the small deviation from the predicted probabilities.

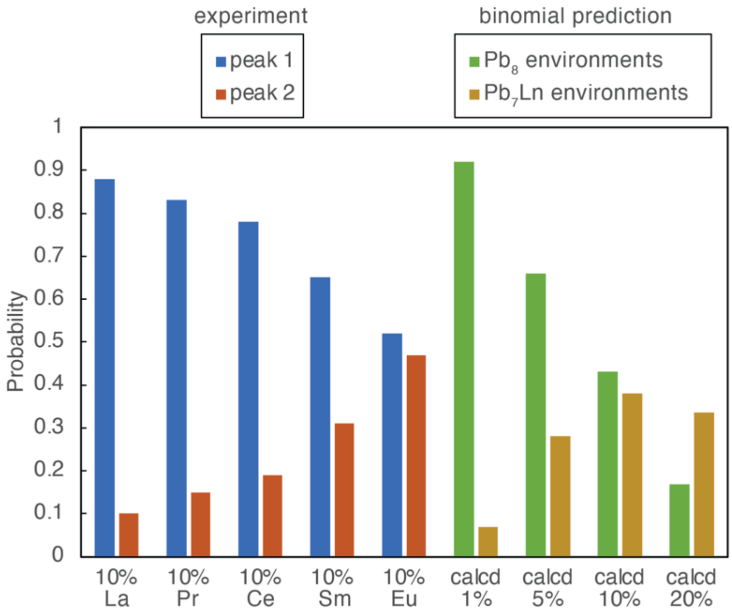


Figure S19. Comparison of (left part) the experimentally measured areas of the two most intense peaks in the quantitative $^{133}Cs$ spectra of $CsPbCl_3$ doped with $LaCl_3$, $PrCl_3$, $CeCl_3$, $SmCl_3$, and $EuCl_3$ and (right part) the probabilities of occurrence of different local Cs environments calculated using the binomial theorem. The two local Cs environment shown here are: Cs surrounded by 8 Pb atoms ($Pb_8$), and Cs surrounded by 7 Pb and 1 L atoms ($Pb_7Ln$). The experimental areas were obtained by fitting the spectra. Note that the materials studied here are non-stoichiometric (aliovalent substitution, i.e., $Pb^{2+}$ replaced by $Ln^{3+}$) and the dopants ($LnCl_3$) were added as excess relative to stoichiometric $CsPbCl_3$, which is the likely origin of the deviations from the predicted probabilities. The second major source of discrepancy is likely the peak deconvolution process, especially in the case of Eu.

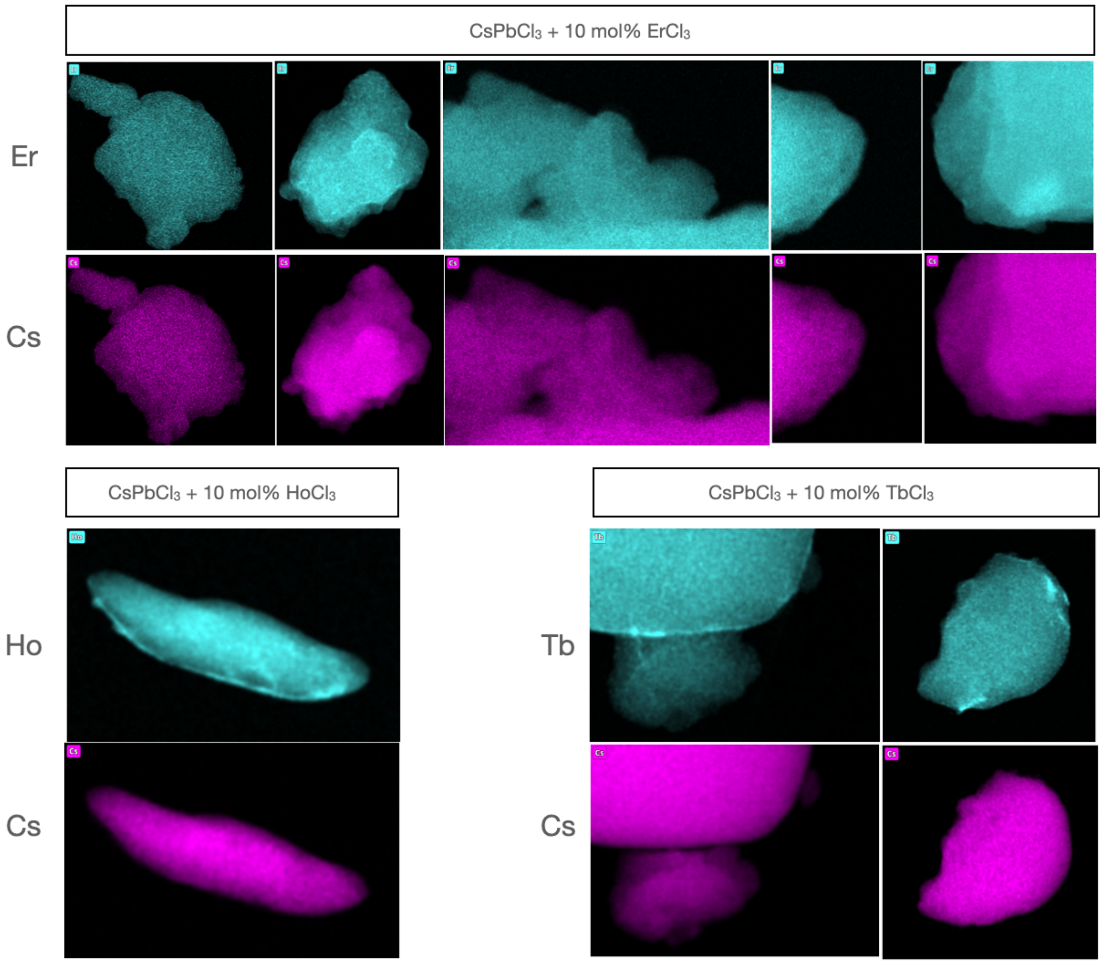


Figure S20. STEM EDX maps of $CsPbCl_3$ doped with $ErCl_3$, $HoCl_3$, and $TbCl_3$. The full maps including Pb and Cl are given in Figure S21 and S22.

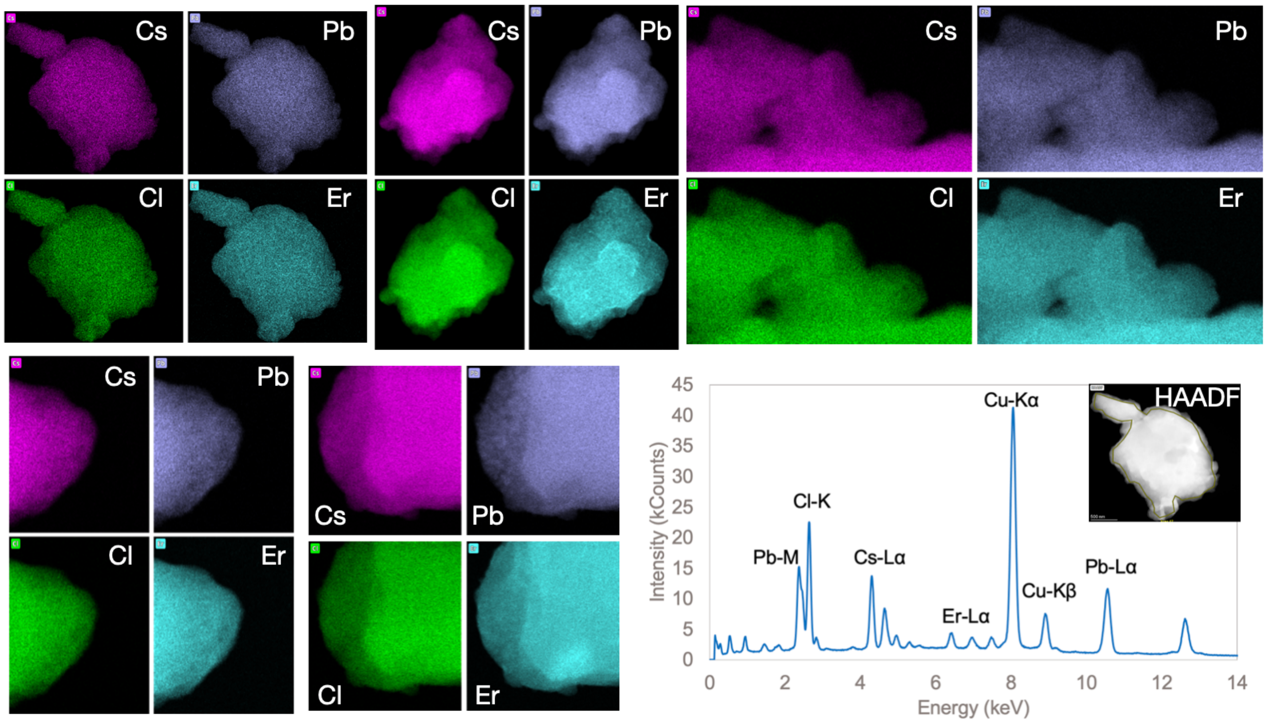


Figure S21. The full STEM EDX maps of $CsPbCl_3$ doped with $ErCl_3$. An example EDX spectrum with the corresponding HAADF image (inset) is also shown.

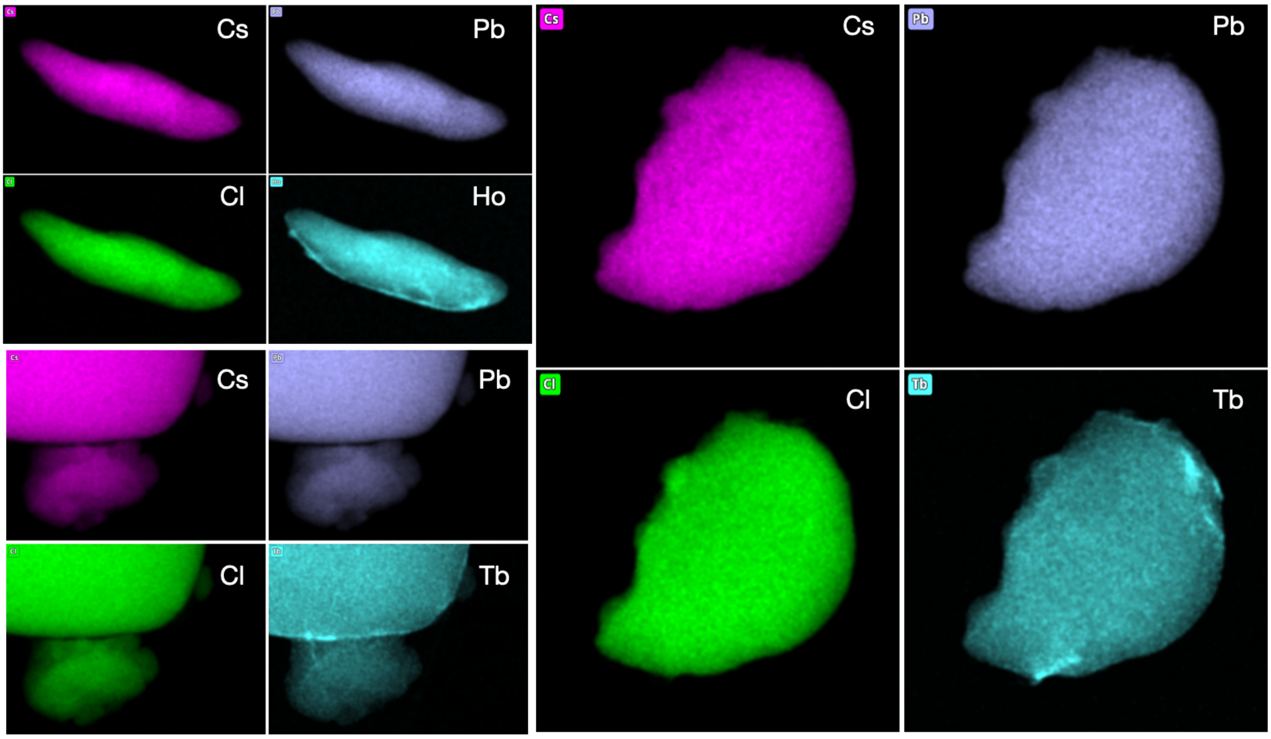

Figure S22. The full STEM EDX maps of $CsPbCl_3$ doped with $HoCl_3$ and $TbCl_3$.

## Supplementary Note 1: the binomial theorem.

*The binomial theorem*. The random distribution of x atoms of species X and y atoms of species Y on N sites is given by the following formula:

$$P = \frac{N!}{n!(N-n)!}(p_x)^n(1-p_x)^{N-n}$$

where $p_X$ represents the fraction of species X and $1-p_X$ is the fraction of species Y in the sample. In the specific case of $Cs^+$ surrounded by eight $Pb^{2+}$ nearest neighbours, there are 8 surrounding $Pb^{2+}$ sites, with X = $Pb^{2+}$ and Y=$Ln^{3+}$. Index *n* takes on values from 0 to N. n=8 corresponds to a local $Cs^+$ environment surrounded by 8 $Pb^{2+}$; n=7 corresponds to a local $Cs^+$ environment surrounded by 7 $Pb^{2+}$ and 1 $Ln^{3+}$. Figures SX and SX plot the binomial predictions for n=8 and 7.

## Supplementary Note 2: Anisotropic bulk magnetic susceptibility (ABMS)

$Er^{3+}$ is a magnetically anisotropic ion. In a polycrystalline powder, every crystallite experiences a demagnetizing field due to its neighbouring crystallites which leads to inhomogeneous broadening of the signals which is not removed by MAS – this is the ABMS effect.[1] While ABMS is expected for other late lanthanides with high anisotropies of the magnetic susceptibility (Tb, Dy, Ho, Er, Tm, Yb), we only observed the extra broadening in doped $CsPbCl_3$ the case of $Er^{3+}$. We carried out additional experiments to corroborate this result. Repeating the synthesis resulted in a material with an essentially identical $^{133}Cs$ NMR spectrum (Fig. S21a). We have also prepared $Cs_3Er_2Cl_9$, an erbium-rich phase to establish the extent of ABMS it exhibits. The spinning sidebands in its $^{133}Cs$ NMR spectrum are substantially broadened (FWHM of about 2000 Hz) but they exhibit a relatively long $T_2$ of 3 ms, measured in a variable echo delay experiment, which corresponds to a natural line width of about $w_{1/2} = 1/(\pi \cdot 0.003\ s) = 100$ Hz (Fig. S21b). This result shows that the $^{133}Cs$ spectrum of $Cs_3Er_2Cl_9$ is inhomogeneously broadened as a result of large ABMS. This is in stark contrast to other lanthanide-rich solids, such as $CsEuI_3$ and $Cs_3NdCl_6$, whose spectra show much narrower

resonances (Fig. S21b). However, at this time, it is unclear why the other late lanthanides do not exhibit a similar extent of $^{133}Cs$ signal broadening in doped $CsPbCl_3$.

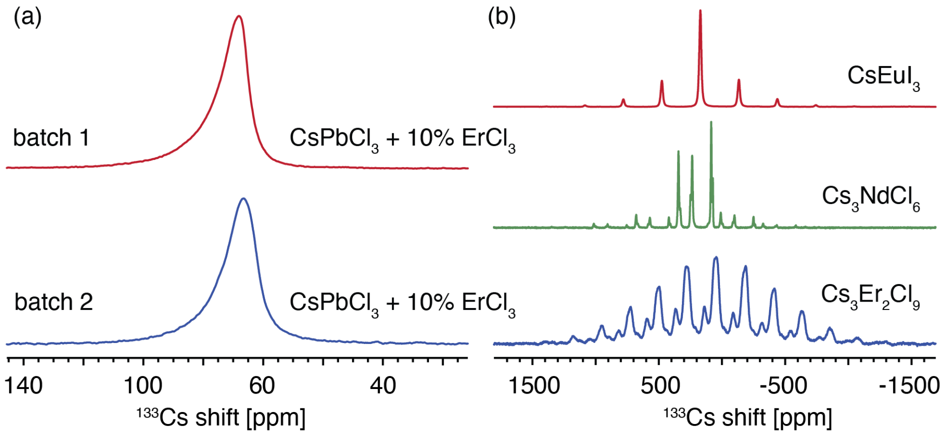


Figure S23. (a) $^{133}Cs$ MAS NMR spectra of two separate batches of $CsPbCl_3$ + 10% $ErCl_3$ (batch 2 was synthesized using different precursor batches than those used for batch 1 to eliminate the possibility of contamination); (b) Comparison of three lanthanide-rich cesium halometalates: $CsEuI_3$, $Cs_3NdCl_6$ and $Cs_3Er_2Cl_9$. The pronounced broadening of spinning sidebands in $Cs_3Er_2Cl_9$ is caused by ABMS.

## Supplementary Note 3: fitting of the saturation-recovery data.

Paramagnetic doping leads to a distribution of relaxation times because PRE is a short-range effect which scales as $1/r^6$, where r is the distance between the nucleus and the paramagnetic species. The curves were therefore fitted using the stretched exponential function:

$$I(\tau) = y_0 + A \cdot e^{-\left(\frac{\tau}{T_1}\right)^{\beta}}$$

where I(τ) is the NMR signal intensity measured after τ seconds of recovery, $y_0$ is an offset accounting for imperfect saturation, $T_1$ is the relaxation time and β is the stretching factor.

The experimental 1D spectra corresponding to different recovery delays were integrated in bins of 1 ppm width (0.23 ppm with for undoped $CsPbCl_3$) and the delay-dependent intensity within each bin was fitted as described above.

## Data fitting results

**undoped $CsPbCl_3$**

$y_0$ = 1.052 (1.051, 1.054)
A = -1.046 (-1.048, -1.045)
$\tau$ = 16.98 (16.91, 17.05)
b = 0.9446 (0.9407, 0.9485)

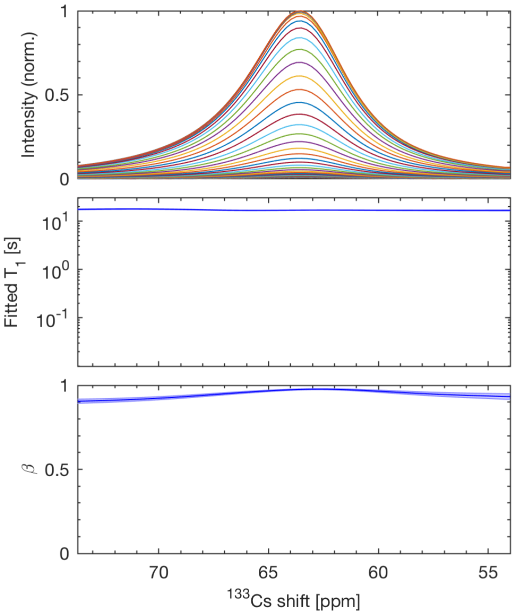


**cerium(III) chloride, 10 mol% excess**

$y_0$ = 1.105 (1.087, 1.122)
A = -1.112 (-1.138, -1.086)
$\tau$ = 1.328 (1.202, 1.454)
b = 0.5229 (0.4906, 0.5552)

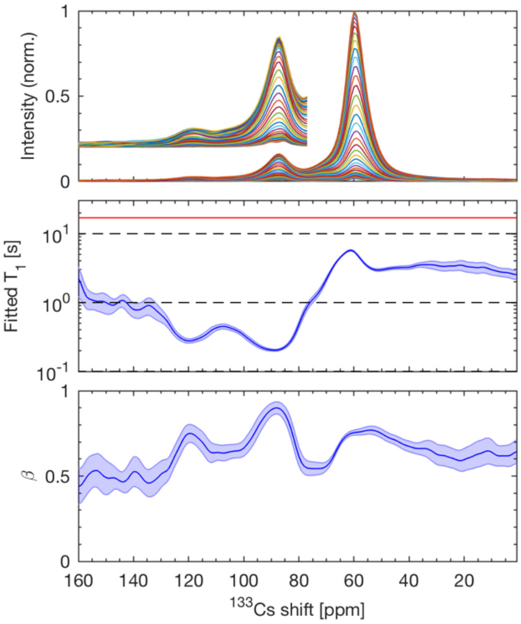

**praseodymium(III) chloride, 10 mol% excess**

$y_0$ = 1.279 (1.255, 1.303)
A = -1.275 (-1.31, -1.241)
$\tau$ = 1.706 (1.52, 1.892)
b = 0.4986 (0.4664, 0.5309)

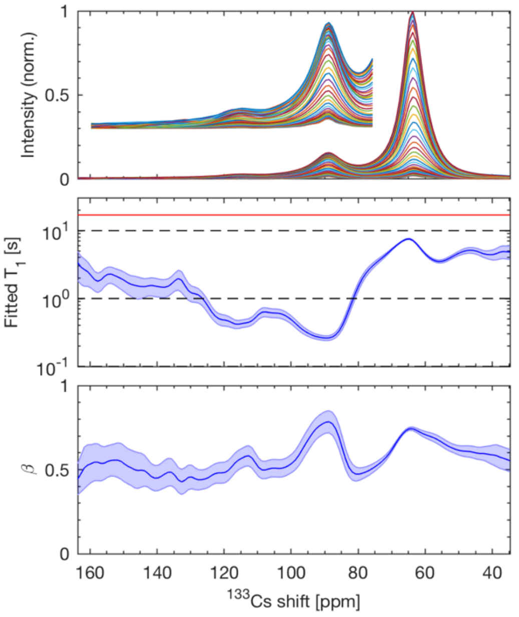


**neodymium(III) chloride, 5 mol% excess**

$y_0$ = 0.992 (0.9779, 1.006)
A = -0.9932 (-1.011, -0.9754)
$\tau$ = 3.396 (3.156, 3.635)
b = 0.5641 (0.5405, 0.5877)

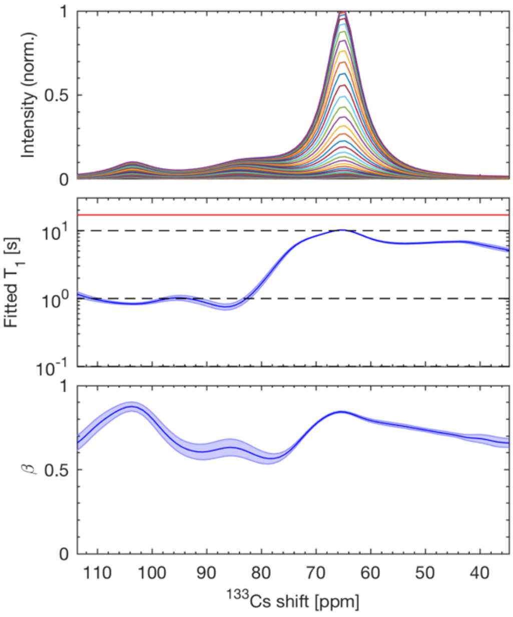


**neodymium(III) chloride, 1 mol% excess**

$y_0$ = 1.099 (1.093, 1.105)
A = -1.097 (-1.103, -1.091)
$\tau$ = 13.37 (13.17, 13.57)
b = 0.8463 (0.8388, 0.8538)

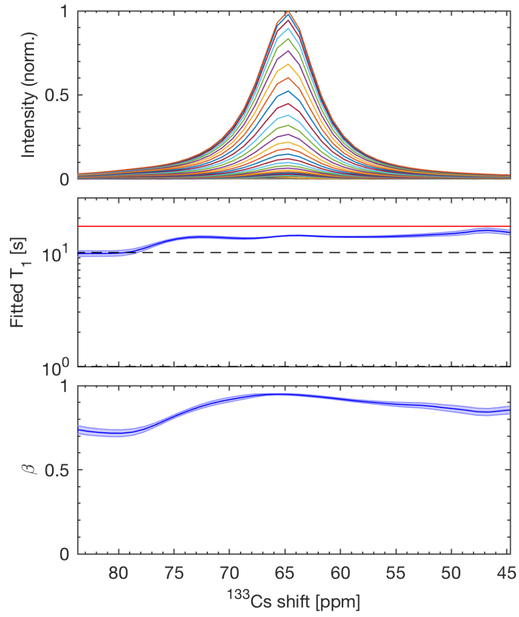


**neodymium(III) chloride, 10 mol% excess**

$y_0$ = 1.04 (1.024, 1.056)
A = -1.015 (-1.042, -0.9883)
$\tau$ = 0.5047 (0.4539, 0.5556)
b = 0.5799 (0.5345, 0.6252)

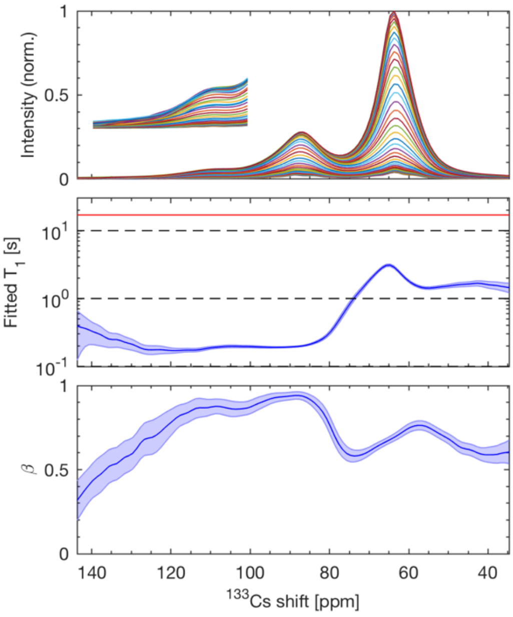

**neodymium(III) chloride, 20 mol% excess**
$y_0$ = 1.024 (1.016, 1.031)
A = -1.009 (-1.02, -0.9985)
$\tau$ = 0.4547 (0.4376, 0.4718)
b = 0.7558 (0.7294, 0.7822)

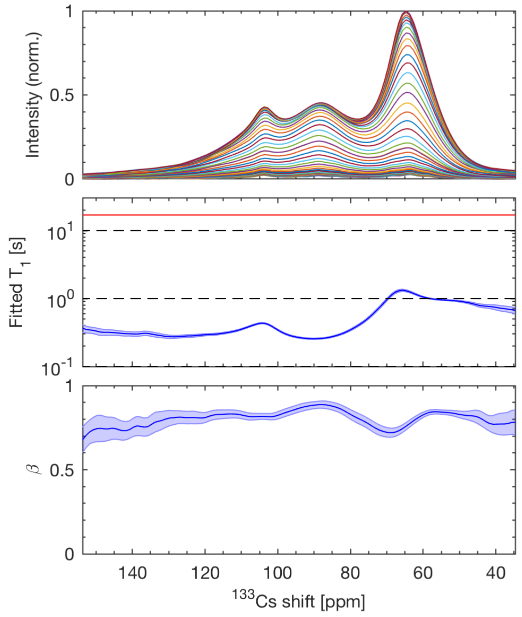


**samarium(II) iodide, 10 mol% excess**
$y_0$ = 1.099 (1.091, 1.108)
A = -1.118 (-1.131, -1.104)
$\tau$ = 0.5938 (0.5675, 0.6201)
b = 0.7064 (0.6787, 0.734)

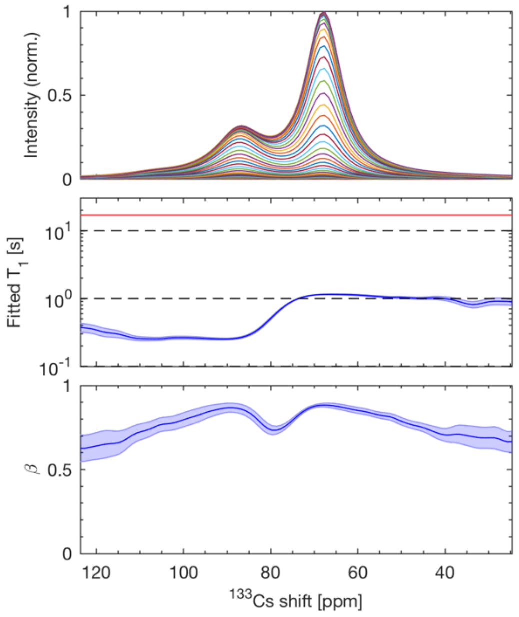


**samarium(III) chloride, 10 mol% excess**
$y_0$ = 1.028 (1.012, 1.045)
A = -1.018 (-1.046, -0.9895)
$\tau$ = 0.6011 (0.5366, 0.6655)
b = 0.5322 (0.4914, 0.5731)

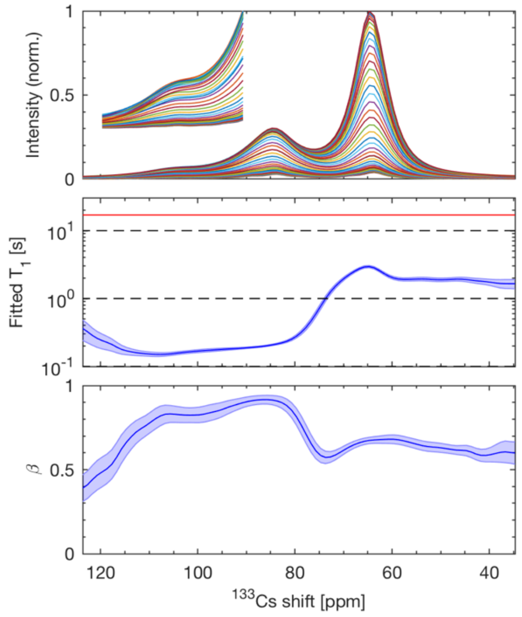


**europium(III) chloride, 10 mol% excess**
$y_0$ = 1.162 (1.154, 1.169)
A = -1.172 (-1.188, -1.157)
$\tau$ = 0.2365 (0.2256, 0.2474)
b = 0.5576 (0.5365, 0.5787)

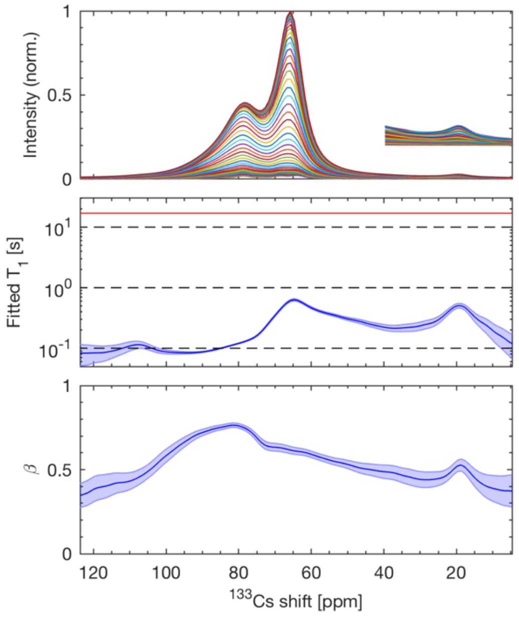

**europium(II) iodide, 10 mol% excess**

$y_0$ = 1.082 (1.075, 1.088)
A = -1.132 (-1.149, -1.114)
$\tau$ = 0.116 (0.1102, 0.1217)
b = 0.4962 (0.4773, 0.5152)

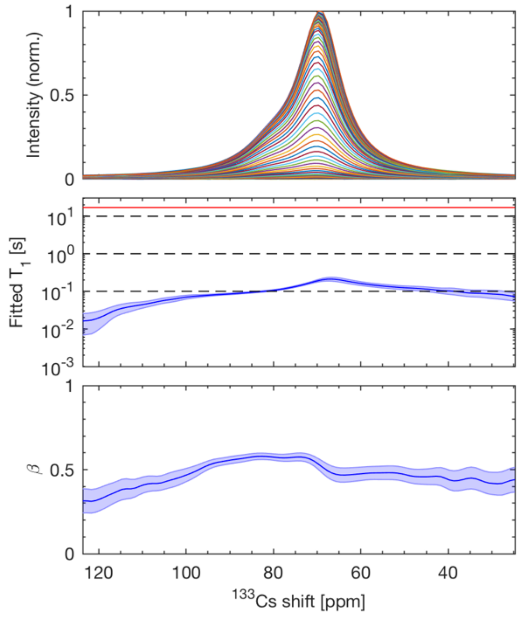


**terbium(III) chloride, 10 mol% excess**

$y_0$ = 1 (0.9824, 1.018)
A = -0.9864 (-1.013, -0.9595)
$\tau$ = 1.121 (0.9984, 1.243)
b = 0.5238 (0.4864, 0.5613)

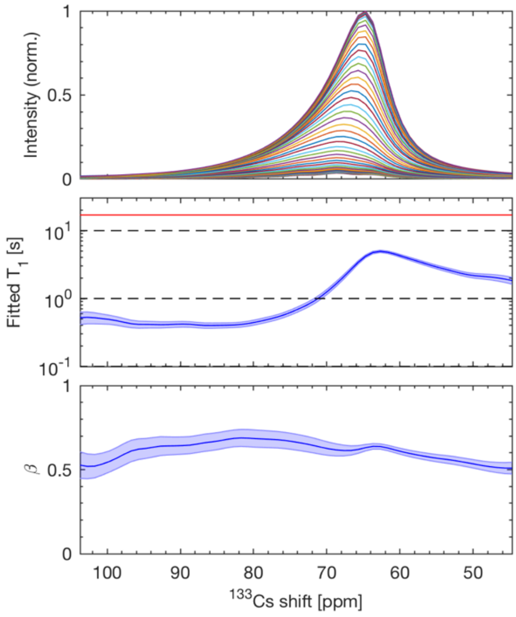


**gadolinium(III) chloride, 10 mol% excess**

$y_0$ = 1.038 (1.022, 1.053)
A = -1.188 (-1.265, -1.112)
$\tau$ = 0.03895 (0.03078, 0.04712)
b = 0.3878 (0.3415, 0.434)

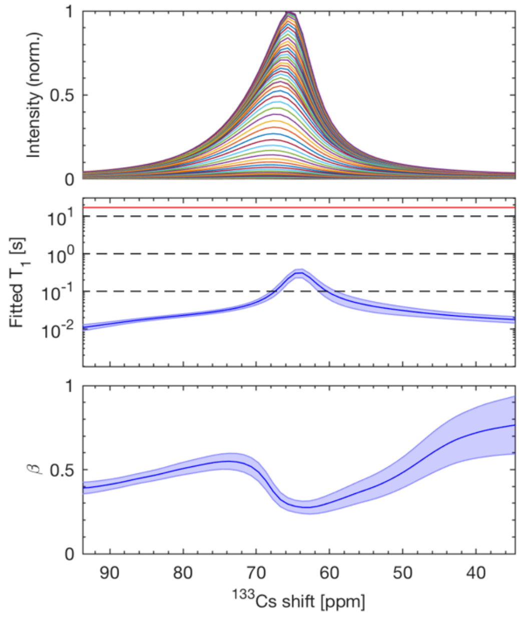


**dysprosium(III) chloride, 10 mol% excess**

$y_0$ = 1.057 (1.029, 1.084)
A = -1.072 (-1.11, -1.035)
$\tau$ = 2.615 (2.239, 2.991)
b = 0.4085 (0.382, 0.435)

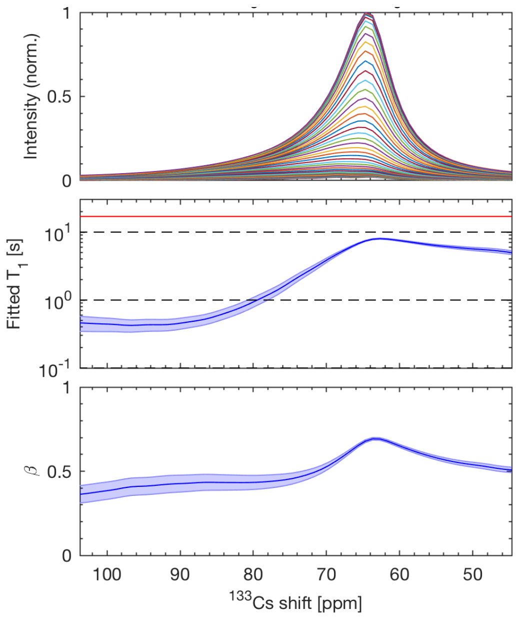

**holmium(III) chloride, 10 mol% excess**

$y_0$ = 0.9876 (0.9683, 1.007)
A = -1.044 (-1.08, -1.008)
τ = 0.7874 (0.6859, 0.889)
b = 0.3599 (0.335, 0.3848)

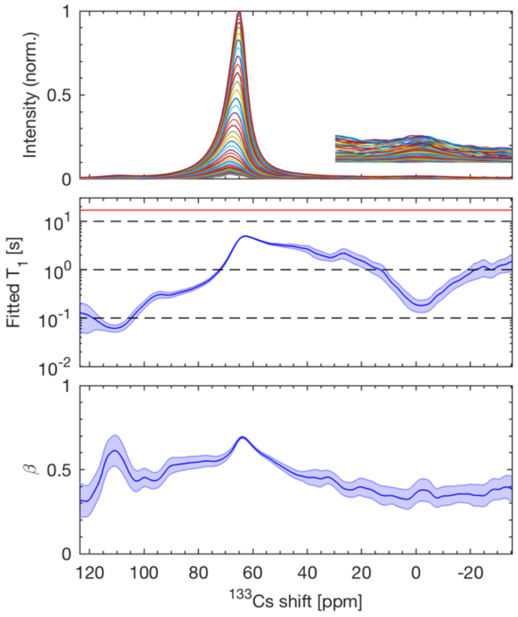


**erbium(III) iodide, 10 mol% excess**

$y_0$ = 1.064 (1.049, 1.08)
A = -1.109 (-1.134, -1.083)
τ = 0.7503 (0.682, 0.8187)
b = 0.4998 (0.4694, 0.5301)

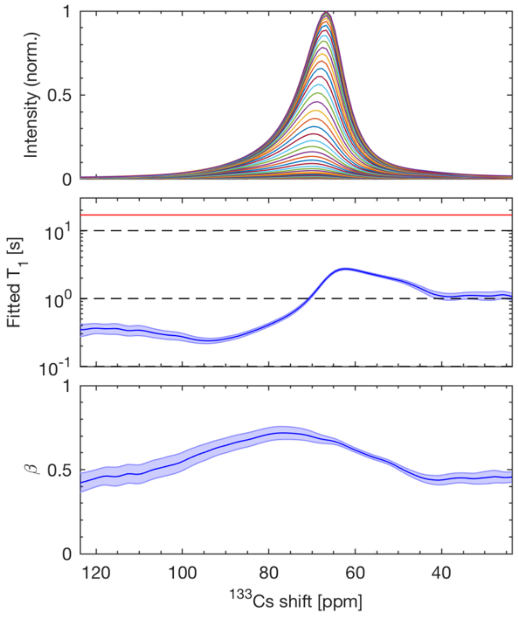


**erbium(III) chloride, 10 mol% excess**

$y_0$ = 1.046 (1.04, 1.052)
A = -1.015 (-1.026, -1.005)
τ = 0.4195 (0.4031, 0.4359)
b = 0.5906 (0.5721, 0.6091)

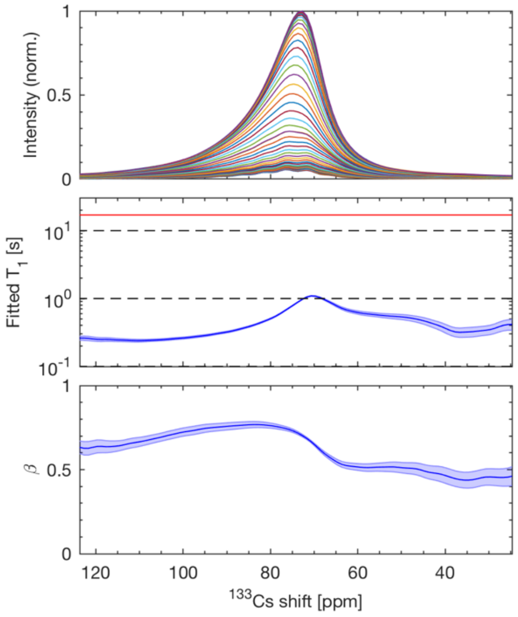


**thulium(III) chloride, 10 mol% excess**

$y_0$ = 1.047 (1.026, 1.068)
A = -1.039 (-1.071, -1.007)
τ = 1.087 (0.9532, 1.22)
b = 0.5233 (0.481, 0.5655)

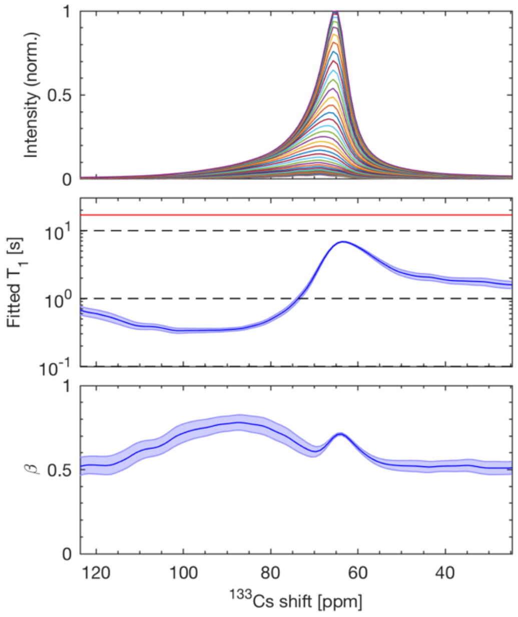

**ytterbium(III) chloride, 10 mol% excess**

$y_0$ = 1.215 (1.198, 1.233)
A = -1.195 (-1.217, -1.173)
τ = 3.57 (3.315, 3.825)
b = 0.5629 (0.5393, 0.5865)

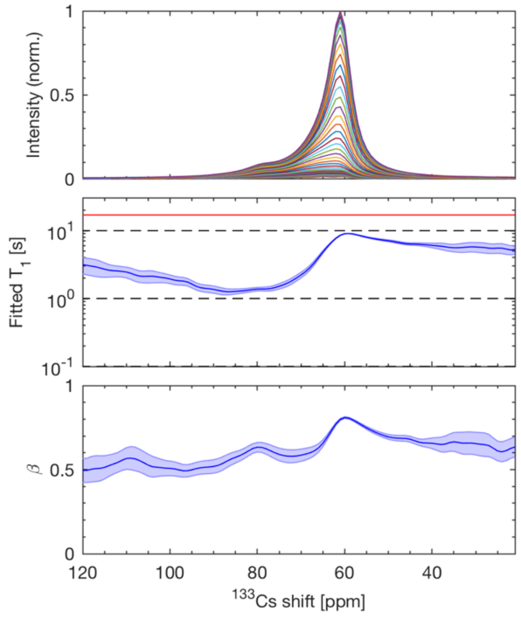


**$Cs_3NdCl_6$**

$y_0$ = 0.9807 (0.9763, 0.985)
A = -0.9641 (-0.9695, -0.9587)
τ = 0.6546 (0.6433, 0.666)
b = 0.9559 (0.9386, 0.9732)

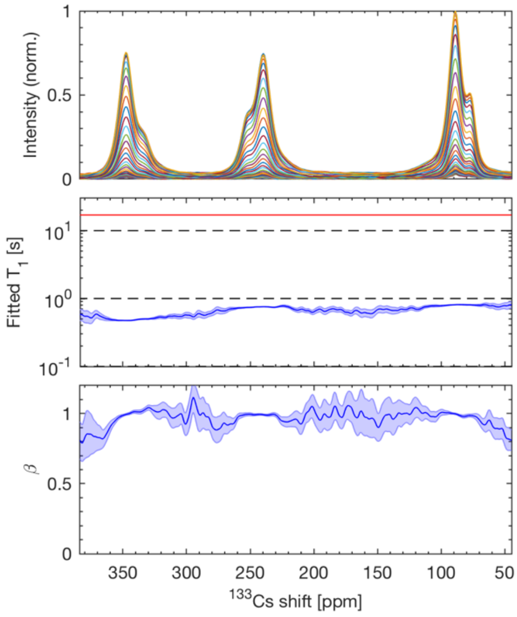


## References

(1) Pell, A. J.; Pintacuda, G.; Grey, C. P. Paramagnetic NMR in Solution and the Solid State. *Prog. Nucl. Magn. Reson. Spectrosc.* **2019**, *111*, 1–271. https://doi.org/10.1016/j.pnmrs.2018.05.001.
